# Biophysical modeling of thalamocortical circuit dynamics: species-specific insights into neural synchrony, sleep spindles, and circuit mechanisms

**DOI:** 10.1093/sleepadvances/zpag100

**Published:** 2026-09-04

**Authors:** Basilis Zikopoulos, Natalia Matuk, Irina Romanova, Arash Yazdanbakhsh

**Affiliations:** Graduate Program for Neuroscience, Boston University, Boston, MA, United States; Center for Systems Neuroscience, Boston University, Boston, MA, United States; Human Systems Neuroscience Laboratory, Department of Health Sciences, Program in Human Physiology, Boston University, Boston, MA, 02215 United States; Department of Anatomy and Neurobiology, Boston University School of Medicine, Boston, MA, United States; Human Systems Neuroscience Laboratory, Department of Health Sciences, Program in Human Physiology, Boston University, Boston, MA, 02215 United States; Computational Neuroscience and Vision Laboratory, Department of Psychological and Brain Sciences, Boston University, Boston, MA, 02215 United States; Human Systems Neuroscience Laboratory, Department of Health Sciences, Program in Human Physiology, Boston University, Boston, MA, 02215 United States; Computational Neuroscience and Vision Laboratory, Department of Psychological and Brain Sciences, Boston University, Boston, MA, 02215 United States; Graduate Program for Neuroscience, Boston University, Boston, MA, United States; Center for Systems Neuroscience, Boston University, Boston, MA, United States; Computational Neuroscience and Vision Laboratory, Department of Psychological and Brain Sciences, Boston University, Boston, MA, 02215 United States

**Keywords:** core thalamocortical circuits, matrix thalamocortical circuits, rodents, primates, closed, open, and hybrid loop thalamocortical connectivity, core and matrix thalamocortical loop, neural activity synchronization, sleep spindles

## Abstract

Thalamocortical circuits play a central role in sensory processing, attention, and sleep spindles. Here, we used a biophysically-grounded computational model with single-compartment neurons and species-inspired thalamocortical architectures to investigate how core and matrix pathways, thalamic interneurons, and open, closed, or hybrid thalamic reticular nucleus–thalamic loop configurations influence synchrony, spatiotemporal signal propagation, and spindle-like activity. The model incorporated both core versus matrix thalamocortical projections, with core pathways providing focal, driving input to middle cortical layers and matrix pathways providing more widespread, modulatory signaling across superficial layers, along with pathway-specific receptor dynamics and rodent-like versus primate-like circuit configurations that differed primarily in the presence of thalamic interneurons, as these cells are sparse in rodents but comprise up to a third of the thalamic neurons in primates. Across the tested conditions, the rodent-like circuits were more sensitive to parameter changes in core and matrix thalamocortical connectivity strength, while primate-like circuits maintained relatively stable spatiotemporal patterns across parameter variations, exhibiting greater stability and synchrony. Spindle analyses further suggested greater variability in event structure and timing in rodent-like networks and more regular spindles in primate-like networks. These findings provide insights into how species-inspired architectural differences may shape thalamocortical dynamics, thereby affecting spindle generation and network synchronization.

**Statement of Significance**

Thalamocortical circuits generate sleep spindles and support sensory processing and attention; yet, how pathway-specific architecture and species differences shape spindle dynamics is poorly understood. Using a biophysically grounded model that contrasts core versus matrix thalamocortical projections, includes thalamic interneurons, and tests TRN–thalamic loop configurations, we find rodent-like networks are more parameter-sensitive with variable spindle structure, whereas primate-like networks, enriched with thalamic interneurons, show greater stability, synchrony, and spindle regularity. These results identify mechanistic circuit features that can explain species differences in spindle generation and highlight gaps in linking microcircuit architecture to sleep physiology. The findings yield testable predictions for electrophysiology and imaging and caution that species-specific motifs matter when translating spindle research to human sleep disorders.

## Introduction

The thalamocortical (TC) circuit, composed of the cortex, thalamus, and the inhibitory thalamic reticular nucleus (TRN), plays a critical role in both wake and sleep states [1]. During wakefulness, the TC circuit is involved in sensory processing and attention [1–3], while during sleep, it generates sleep spindles, which are brief oscillations in the ~10–16 Hz range during non-rapid eye movement (REM) sleep, commonly detected via electroencephalogram (EEG) recordings, and facilitates memory consolidation [4, 5]. Altered spindle activity has also been implicated in neuropsychiatric and neurodevelopmental disorders, including schizophrenia (SCZ) and autism, with prior studies linking these conditions to disrupted coordination of non-REM sleep oscillations and TRN dysfunction, thus motivating investigation of the circuit mechanisms that underlie sleep spindle generation and synchrony [5–8].

In an influential framework, a major organizational principle of the TC system is the presence of two parallel and distinct sub-circuits: the core and the matrix [9]. Core circuits are involved with sensory and cognitive processing and consist of focal projections to middle cortical (Ctx) layers, whereas matrix circuits are involved with limbic processing and memory consolidation, consisting of diffuse and widespread projections to superficial Ctx layers [10]. Other complementary frameworks for TC organization, such as driver/modulator, and first-order/higher-order distinctions, have also been proposed [11–15]. Core and matrix pathways are found across mammals, although key differences exist between species. In primates, the significant expansion and specialization of the thalamus and of Ctx areas without clear rodent homologs are associated with a clearer separation of core and matrix TC circuits [16–24]. By contrast, rodents differ in the laminar distribution of Ctx and thalamic projection neurons, their termination patterns, and their neurochemical profiles in thalamus [25–28]. Moreover, rodents possess relatively few thalamic interneurons (IN) restricted to specific thalamic nuclei, whereas in primates, IN make up approximately a third of all thalamic neurons [29]. This interspecies variability makes it crucial to examine TC dynamics across both rodent-like and primate-like circuit architectures.

Another important distinction lies in the organization of TRN–thalamic interactions. These can be arranged in closed-loop (reciprocal innervation between TRN and the same thalamic neurons they inhibit), open-loop (no such reciprocity), or hybrid configurations that combine features of both. Despite extensive research on the role of the TC circuit in awake processing and sleep spindle generation, the specific contributions of core versus matrix circuits, TRN–thalamic connectivity configurations, and local inhibition, remain incompletely understood, particularly in relation to how these factors may contribute to species-related functional differences.

To address this gap, we developed a biophysically-grounded conductance-based computational model of TC circuitry that incorporates species-informed structural and functional properties. We refer to these models as rodent-like and primate-like because they capture specific architectural features informed by selected interspecies anatomical differences. These models are intentionally simplified and are designed to isolate the effects of selected organizational features, most notably the presence or absence of thalamic IN. By using simplified single-compartment neurons and circuit architectures, the model allows us to isolate how selected circuit design features influence network dynamics in rodent-like and primate-like configurations. Thus, the goal is to examine how specific architectural differences may influence synchrony, spatiotemporal recruitment, and spindle-band activity in a controlled computational modeling framework.

The model includes core, matrix, and mixed circuits, with the mixed circuit representing a functional combination of the two via cortico–cortico connections. For each species-inspired model, we simulated across closed, open, and hybrid loop configurations. The hybrid loop represents a distinct loop configuration that integrates the features of both open and closed architectures. The hybrid loop was included because purely open and purely closed architectures likely represent limiting cases, whereas biological thalamoreticular circuitry may contain both motifs [3, 30–47]. Based on our prior work [48], the hybrid design provides a framework for representing intermediate TRN–thalamic organization and testing whether mixed loop structure produces dynamics distinct from either extreme. We also incorporated distinct and opposite receptor dynamics for each circuit: in the core, ionotropic receptors mediated TC connections and metabotropic receptors mediated corticothalamic connections, whereas in the matrix the arrangement was reversed. We controlled the spatial spread of neural activity using a difference-of-Gaussians convolution, with a narrower filter for the focal projections of the core circuit and a wider filter for the diffuse projections of the matrix circuit. Because these receptor and spatial differences were implemented together, the present simulations do not isolate their individual contributions to spindle structure or synchrony.

Network activity was initiated by external input to a single thalamic or TRN neuron, which evoked TRN bursting and subsequent rebound spiking in TC neurons. This interaction initiated spindle-like activity, which we characterized by averaging multi-neuron activity and applying a 9–13 Hz bandpass filter to extract spindle signals. Using these models, we tested how species-informed differences in interneuron content, core/matrix organization, and TRN–thalamic loop architecture shape synchrony, spatiotemporal aspects of neural signals, and spindle-like dynamics. Our simulations showed faster synchronization, as well as higher spindle density and amplitude in rodent-like than in primate-like networks. They also suggested distinct forms of spindle vulnerability: spindle activity in core circuits was more vulnerable to TC manipulations overall, while matrix manipulations were more effective at reducing overall spindle density.

## Materials and methods

### TC anatomy and connectivity

**Figure 1:**
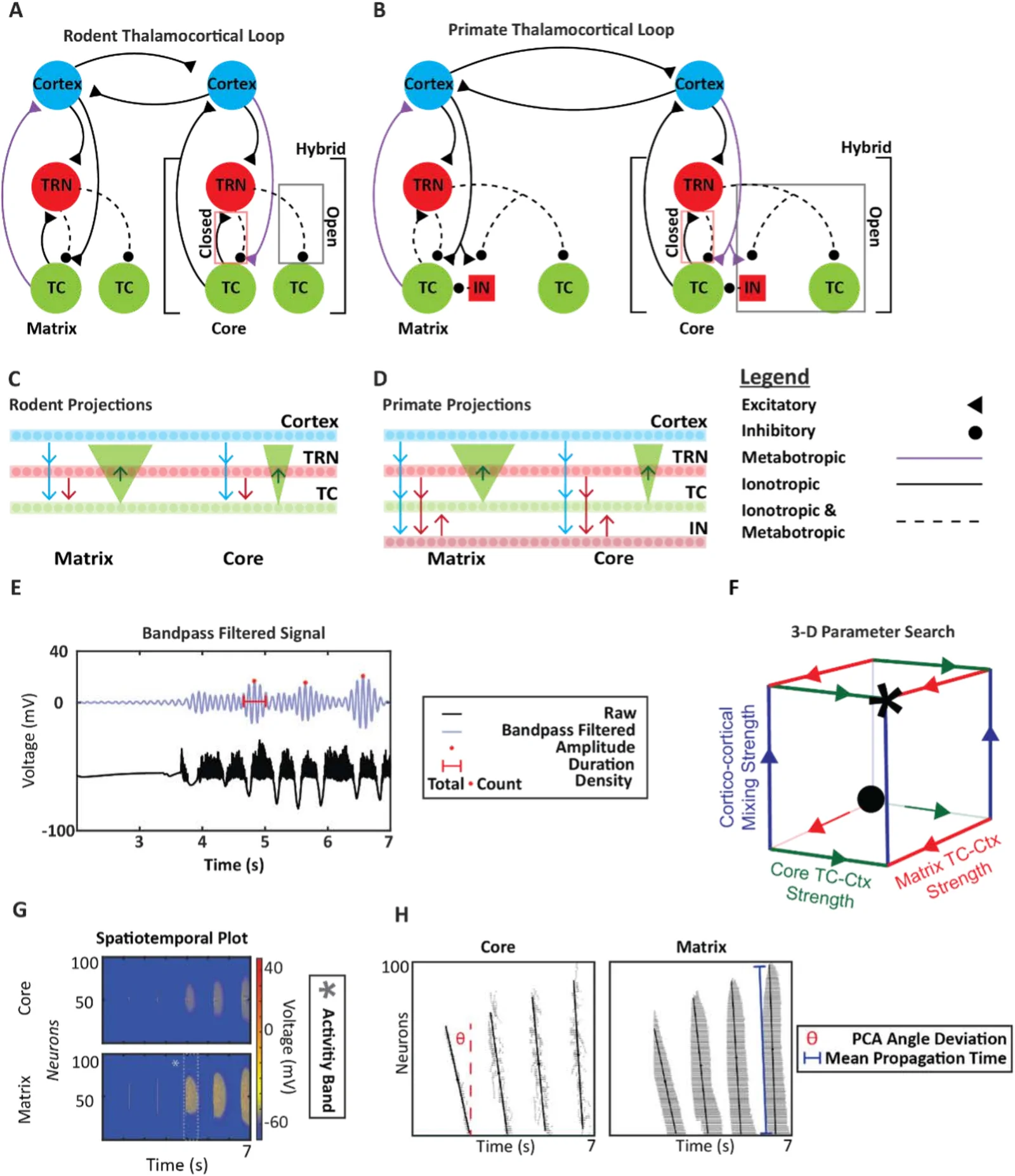
Experimental design and TC loop connectivity. (A and B) The rodent-like and primate-like TC model. The Ctx, TC relay, and TRN neurons (circles) make up the TC rodent-like and primate-like model loops. Local thalamic inhibitory IN (square) are an additional component of the primate-like circuitry. The different types of synapses and receptors mediating the connections are labeled in the legend. GABA_A_ (ionotropic) or GABA_B_ (metabotropic) mediate inhibitory synapses. AMPA (ionotropic) or mGluR (metabotropic) mediate excitatory synapses. The diagrams indicate the modeled TC loop configurations: closed, open, and hybrid. The closed loop is defined as thalamic reticular neurons inhibiting thalamic relay cells that reciprocally excite them. The open loop is defined as thalamic reticular neurons inhibiting thalamic relay cells that do not reciprocally excite them. The hybrid loop is defined as a combination of the open and closed loop. (C and D) Rodent-like and primate-like projections. Connectivity was implemented at the population level using pathway-specific spatial kernels, as summarized in Tables S5 and S6. TC projections differed in spatial spread between core and matrix pathways, with core projections implemented more focally and matrix projections implemented more diffusely. The matrix TC projections were an order of magnitude wider than the core TC projections through the use of Gaussian filters and convolution. (E) Sleep spindle detection. The top (light) trace is the bandpass-filtered signal with the frequency range of 9–13 Hz and the bottom (dark) trace is the averaged neural activity. The attributes of spindles that were of interest are present in the legend. (F) The 3D cube template represents the 3D parameter search of core and matrix TC mixing strength and cortico–Ctx mixing strength. The * indicates the control case and the • indicates the starting case. (G) Sample spatiotemporal plot of core and matrix Ctx activity. The dashed gray box highlights an activity band. (H) Raster plot of core and matrix Ctx activity, with a PCA-derived eigenvector overlaid on each raster plot. Its deviation from vertical (θ) measures synchrony, and the distance between its endpoints is used to compute the MPT, quantifying temporal propagation across neurons.

The TC model includes Ctx, TC relay, and TRN neurons in the rodent-like and primate-like models; the latter also contains local thalamic inhibitory IN (Figure S1). Core and matrix circuits interact at the Ctx level, incorporating inter- and intra-Ctx area mixing. Thalamic microarchitectural differences have been debated previously and implemented into this simulation [30, 40, 48]. Our model contains three different configurations: closed, open and hybrid loop to understand how it impacts the activity of the circuit as a whole (Figure 1A and B). The closed loop refers to a configuration in which the TRN neuron inhibits the thalamic relay (TC) neuron, which reciprocally excites the original TRN neuron. In contrast, the open loop describes a configuration where the TRN neuron inhibits a TC neuron without reciprocal excitation. The hybrid loop combines features of both closed and open configurations [28]. The present implementation follows the same general hybrid-loop framework introduced in our previous work, in which spatially structured TRN–thalamic connectivity can serve as an intermediate state between functionally open and closed organization [48]. Because empirical data on thalamoreticular connectivity remain limited, with existing anatomical studies suggesting that both open and closed motifs coexist within the TC system, a purely open or purely closed design would fail to capture the full range of biologically plausible dynamics. Incorporating a hybrid loop therefore provides an approximation of real-world circuitry, allowing us to simulate interactions that likely occur in vivo.

Core and matrix circuits differ in their neurochemical and neuroanatomical properties. Based on anatomical and physiological findings [23, 32], NR1 ionotropic receptors mediate matrix Ctx–TC synapse, while mGluR1a metabotropic receptors mediate core Ctx–TC synapse [25, 49]. These receptor type differences were implemented in our model (Figure 1C and D). For the reciprocal TC connections, receptor types were reversed to maintain their driving versus modulatory roles of the core and matrix pathways, with driver synapses referring to inputs that convey the principal signal and modulatory synapses referring to inputs that regulate that transmission [11]. Furthermore, the core and matrix TC projections differ in their target regions and spread. Matrix TC projections target the superficial Ctx layers in a widespread manner, while core TC projections target the middle/deep Ctx layers more focally [9, 25]. Gaussian filters and convolution were used to simulate these differences, with matrix projections having a spread an order of magnitude wider than core projections.

### Network geometry

The reduced rodent-like network contained six neuronal populations, whereas the expanded primate-like network contained eight populations by including thalamic interneuron populations in both core and matrix pathways. Each neural population was represented by the same number of neurons (100 neurons per neural type). Thus, the rodent-like network contained 600 neurons and the primate-like network contained 800 neurons. The models simulated connections between populations and not within populations. The models did not explicitly encode biological cell-count ratios by assigning different biological values. Instead, relative neuron count influence was approximated through synaptic conductance scaling to reflect differences in effective connectivity between neural populations. Connectivity was defined at the population level according to modeled circuit architecture and was not limited to one-to-one cell projections (Figure 1A and B). All neurons were modeled as single-compartment units governed by first-order differential equations that incorporate voltage-gated, intrinsic and synaptic currents. The simulation activity was initiated through a brief (50 ms) pulse to the circuit, and each simulation of the model ran for 7 s.

Within each population, neurons were arranged along a one-dimensional spatial axis indexed from 1 to 100. Projections were topographically organized using population-specific spatial kernels, such that each presynaptic neuron influenced a local neighborhood of postsynaptic targets according to the projection-specific spread function, as summarized in Tables S5 and S6. Core TC projections were implemented with a narrow Gaussian profile, facilitating focal connectivity, whereas matrix projections used a broader Gaussian profile, producing a more diffuse connectivity. Cortico–Ctx mixing between core and matrix Ctx populations was implemented using the same spatial principle, with connection strength set by the mixing parameter. Conductances for core and matrix were not normalized to preserve equal total projection weight across kernels; instead, pathway strength was allowed to differ in total integrated magnitude across projections with different spatial spreads. Thus, the effective influence of a pathway reflected the combined effects of its conductance and its spatial spread.

### Parameter variation

The three-dimensional (3D) parameter search was implemented as a structured sweep over three parameter families. The *x*- and *y*-axes represent the relative prevalence (strength) of core and matrix TC (TC → Ctx) connectivity, respectively, while the *z*-axis represents the strength of cortico–Ctx mixing. In practice, simulations were organized hierarchically by cortico–Ctx mixing condition, then by matrix TC → Ctx parameter setting, and then by core TC → Ctx parameter setting, such that each point in the parameter cube corresponded to an explicit saved simulation condition. The initial and control cases were selected empirically to represent low-activity and balanced-activity conditions, respectively, rather than to reproduce a physiologically calibrated absolute baseline shared across rodent-like and primate-like configurations. The initial and control conductance-scaling values used for each parameter-cube axis are reported in Table S9. The parameter cube should therefore be interpreted as a structured exploration of relative connectivity scaling within the model rather than a direct mapping onto fixed biological conductance levels.

### Voltage-gated, intrinsic, and synaptic currents

The dynamics of each neuron are described by first-order differential equations based on the original work by Hodgkin and Huxley [50], with adaptations from Pospischil et al. [51] to capture additional physiological features. Each neuron incorporates Hodgkin–Huxley equations to describe sodium (I_Na_), potassium (I_K_) and leak (I_L_) currents, along with intrinsic currents specific to the physiological properties of the neural type and the synaptic currents. All currents were modeled based on Hodgkin–Huxley kinetics. All the relevant equations and constant values are listed in Tables S1 and S2.


$${C}_m\frac{dV}{dt}=-{g}_{leak}\left(V-{E}_{leak}\right)-{I}_{Na}-{I}_K-{I}_{Intrinsic}-{I}_{syn}$$

Each voltage-gated and intrinsic current has the following format: [51]


$${I}_j=g_j\ast{m}^M\ast{h}^N\ast \left(V-{E}_j\right)$$

The maximal conductance of each current is denoted as *g_j_*. The gating variables *m* and *h* are probabilistic values that control the activation and deactivation kinetics, respectively. The driving force for the current is expressed as (*V – E_j_*), where *E_j_* is the reversal potential.

### Hodgkin–Huxley currents

The Hodgkin–Huxley consists of three primary currents: the voltage-dependent Na^2+^ current, the voltage-dependent K^+^ “delayed-rectifier” current, and the leakage current [50]. The equations for all three currents are provided below and all necessary functions and constants are provided in Tables S1 and S2.

### Sodium current [51]


$${I}_{Na}=g_ {Na}\ast{m}^3\ast h\ast \left(V-{E}_{Na}\right)$$

$$\frac{dm}{dt}={\alpha}_m(V)\left(1-m\right)-{\beta}_m(V)m$$

$$\frac{dh}{dt}={\alpha}_h(V)\left(1-h\right)-{\beta}_h(V)h$$

### Potassium “delayed-rectifier” current [51]


$${I}_K=g_K\ast{n}^4\ast \left(V-{E}_K\right)$$

$$\frac{dn}{dt}={\alpha}_n(V)\left(1-n\right)-{\beta}_n(V)n$$

### Leakage current


$${I}_L=g_L\ast \left(V-{E}_L\right)$$

### Intrinsic currents

To capture the distinct physiological properties of TRN and TC relay neurons, two additional currents were incorporated into the model. Both neuron types possess a low-threshold Ca^2+^ current (I_T_) that is responsible for their bursting and rebound bursting properties; however, the kinetics of this current differ between the TRN and TC neurons, thus requiring distinct equations [52–55]. In addition, TC relay neurons possess a hyperpolarization-activated current (I_H_), which facilitates the repolarization of the TC membrane potential following hyperpolarization [54, 56]. All constants and necessary functions for each current are listed in Tables S1 and S2.

### TRN T-current [57]


$${I}_T=g_{Ca}\ast{m}^2\ast h\ast \left(V-{E}_{Ca}\right)$$

$$\frac{dm}{dt}=\frac{1}{\tau_m(V)}\left[m-{m}_{\infty }(V)\right]$$

### TC relay neuron T-current [52]


$${I}_T=g_{Ca}\ast{m}^3\ast h\ast \left(V-{E}_{Ca}\right)$$

$$\frac{dm}{dt}=\frac{1}{\tau_m(V)}\left[m-{m}_{\infty }(V)\right]$$

### TC relay neuron H-current [52]


$${I}_H=g_H\ast \left({S}_1+{S}_2\right)\ast \left({F}_1+{F}_2\right)\ast \left(V-{E}_H\right)$$

$$\frac{d{S}_1}{dt}={\alpha}_S(V)\ast \left(1-{S}_1-{S}_2\right)-{\beta}_S(V)\ast{S}_1+{k}_2\ast \left[{S}_2-C\ast{S}_1\right]$$

$$\frac{d{F}_1}{dt}={\alpha}_F(V)\ast \left(1-{F}_1-{F}_2\right)-{\beta}_F(V)\ast{F}_1+{k}_2\ast \left[{F}_2-C\ast{S}_1\right]$$

### Synaptic currents

Synaptic communication in the model incorporates the following neurotransmitter receptors: AMPA, NMDA, GABA_A_, GABA_B_, and metabotropic glutamate receptor (mGluR). These ionotropic and metabotropic receptors mediate the synaptic connections.

### Ionotropic channels

The current for ionotropic receptors is modeled using the following equation:


$${I}_{Receptor}=g_{Receptor}\ast{m}_{Receptor}\ast \left(V-{E}_{Receptor(ion)}\right)$$

The maximal conductance, g_receptor_, of the ion associated with the specific receptor; the total fraction of open post-synaptic receptors, m_receptor_, and the driving force (V- E_receptor (ion)_) control the flow of the ions through the specific receptor. The fraction of open receptors is dependent on the concentration of neurotransmitter present. The generic differential equation describing this relationship is as follows:


$$\frac{d{m}_{Receptor}}{dt}={\alpha}_{Receptor}\ast \left[{T}_{Receptor}\right]\ast \left(1-{m}_{Receptor}\right)-{\beta}_{m_{Receptor}}\ast{m}_{Receptor}$$

The parameters *α* and *β* represent the forward and backward binding rates, respectively, while [T_receptor_] represents the concentration of neurotransmitter present. All of the constants associated with each ionotropic receptor are present in Table S3.

### Metabotropic channels

The format for the metabotropic receptor and all necessary equations are provided below (Destexhe, Mainen, et al., 1998). All the parameter values are provided in Table S4. The general current equation is provided below:


$${I}_{Receptor}=g_{Receptor}\ast \frac{{s_{Receptor}}^n}{{s_{Receptor}}^n+{K}_d}\ast \left(V-{E}_{Receptor}\right)$$

The variable, $\frac{{s_{Receptor}}^n}{{s_{Receptor}}^n+{K}_d}$, represents the concentration of second-messengers. The equation for the rate of change of the openness of the post-synaptic receptors is as follows:


$$\frac{d{r}_{Receptor}}{dt}={K}_1\ast \left[{T}_{Receptor}\right]\left(1-{r}_{Receptor}\right)-{K}_2\ast{r}_{Receptor}$$

The variables, K_1_ = 1.3, and K_2_ = 0.006, are constant values similar to the forward and binding rates. An additional third differential equation describes the receptor desensitization rate:


$$\frac{d{s}_{Receptor}}{dt}={K}_3\ast \left({r}_{Receptor}\right)-{K}_4\ast{s}_{Receptor}$$

The sensitization and desensitization rates are represented by the variables K_3_ = 0.09, and K_4_ = 0.0064. The variable, ${s}_{Receptor}$, represents second-messenger concentration that both contributes to the opening of the specific metabotropic receptors and to the decay of second-messenger concentration.

The mGluR, responsible for excitatory signaling, was modeled using the kinetics and kinematics of the GABA_B_ receptor. The reversal potential for mGluR (E_mGluR_) was set to 0 mV, consistent with E_AMPA_.

### TC activity synchronization and statistical analysis

The synchronization of TC activity was assessed both qualitatively and quantitatively. 3D parameter searches were conducted and outlined within a 3D cube framework (Figure 1F). The *x*- and *y*-axes represent the relative prevalence (strength) of core and matrix TC connectivity, respectively, while the *z*-axis represents the strength of cortico–Ctx mixing (Figure 1F). The starting condition of each TC loop iteration (•, Figure 1F) corresponds to the initial state of the loop, where all parameters are set to relatively low values. The control case of the network was defined as a balanced TC activity level, situated between complete inactivity and overly saturated activity, allowing for future investigation of neurobiological parameters.

At each vertex of the cube, spatiotemporal maps for the core and matrix TC activity were displayed, showing activity across neuronal populations, including TRN, TC, IN, and Ctx over time (Figure 1G). To quantify the spatiotemporal structure of TC activity bands, we applied principal component analysis (PCA) to the spike-time raster data for each activity band. Activity bands were included only when they formed a coherent spatiotemporal event with a clear start and end time. Disconnected clusters and partial bands that could not be assigned to one coherent band were excluded. For each activity band, we *z*-scored the time and neuron axes to maintain a shared reference frame across species, loop configurations, and trials. PCA was then performed on the *z*-scored time–neuron activity band to obtain the first principal component (PC1), which reflects the orientation of the largest standard deviation of the activity bands and therefore indicates the orientation of the activity band in the spatiotemporal space. The PCA orientation angle (θ) was computed by projecting the PC1 loading vector onto the raster’s time–neuron plane and measuring its absolute deviation from vertical alignment. Thus, θ values near 0° indicate vertically aligned bands in which neurons fire with minimal temporal offset (high synchrony). Larger θ values indicate tilted, propagating activity in which spiking progresses across neurons over time (lower synchrony).

During analysis of the closed-loop rodent-like model, we observed that most activity bands were bilaterally symmetric about the vertical axis and formed boomerang-shaped patterns. Because PCA extracts the axis of maximum variance, applying PCA to this fully symmetric structure produced an artificially vertical eigenvector, even when the underlying spiking pattern itself was not synchronous. The vertical eigenvector was therefore a geometric artifact of bilateral symmetry, not a reflection of true synchrony. To correct for this artifact and recover the true temporal structure of rodent-like closed-loop activity bands, we performed PCA on the upper half of each activity band only. Removing the symmetric lower half prevents symmetry from forcing PCA-derived eigenvector to align vertically. In this half-band representation, the orientation of the PCA-derived eigenvector reflects the actual spatiotemporal dynamics of the activity band, rather than the imposed geometry of bilateral symmetry. This procedure enabled valid comparisons across rastergrams of rodent-like and primate-like activity, and across closed, open, and hybrid loops. The same global *z*-scoring and angle-based synchrony measures were then applied uniformly across rastergrams.

We further used the PCA PC1 eigenvector orientation to compute the mean propagation time (MPT), defined as the elapsed time between the first and last spiking neurons within an activity band divided by the number of active neurons in that interval. To obtain this value, we embedded the PCA-derived eigenvector as it is stretched across the activity band, allowing us to estimate both the time spanned along this vector with the number of neurons it traversed. MPT therefore measures time offset per neuron in the sequence. Thus, the PCA-derived angle and MPT are related but not identical: the angle describes the geometric orientation of the activity band, whereas MPT expresses that orientation as temporal offset per neuron. Importantly, these measures describe how activity is organized across neurons and time, rather than simply how many neurons are recruited. A larger recruited population can still produce a near-vertical band if neurons fire synchronously, whereas sequential recruitment produces a tilted, propagating band (see also Figure S7). Synchronous activity produces MPT values near zero, whereas propagating traveling wave-like activity yields larger MPT values reflecting lower activity band synchrony. PCA-derived orientation angles and MPT were jointly used to characterize the temporal structure of each activity band. Because PCA-based descriptors are sensitive to band geometry and can be influenced by outliers, we treat angle and MPT as structural descriptors rather than exhaustive measures of synchrony. Future work should complement these measures with correlation-based or phase-based synchrony measures, as used in prior studies of TC spindle coherence and spatiotemporal spindle organization [59, 60], to further test the robustness of the present interpretation.

### Parameter space exploration of IN–TC inhibitory connectivity

To evaluate the effect of thalamic interneuron-mediated inhibition, we performed an additional systematic parameter sweep in the primate-like core–matrix closed-loop model. Specifically, we parametrically scaled the strength of the inhibitory projection from thalamic IN onto TC relay neurons from 0 to 1000 times its baseline model value using logarithmic steps, thereby varying the effective amplitude of the IN-to-TC inhibitory contribution while holding the remaining circuit architecture constant. The primary parameter-cube simulations were run for 7 s, whereas the IN–TC inhibitory-connectivity sweep was run for 10 s to assess the effects of inhibitory scaling over a longer simulation window and to examine late-stage activity. A scaling factor of 1 corresponded to the baseline primate-like parameterization used in this sweep, whereas a scaling factor of 0 removed the GABAergic inhibitory current from thalamic IN onto TC relay neurons while leaving the rest of the primate-like circuit unchanged. Thus, the 0 scaling factor condition isolates one rodent-like architectural feature: the absence of local thalamic interneuron inhibition onto TC relay neurons.

This analysis was intended to test how IN–TC inhibitory connectivity strength shapes synchrony and MPT across the tested parameter space. Across the sweep, activity structure was quantified using the PCA-derived deviation from vertical alignment, θ, and MPT per neuron, MPT, as defined above. The number of active neurons was also provided to contextualize activity patterns under strong inhibitory scaling, because PCA-derived θ and MPT become less reliable when activity is restricted to a small subset of neurons. Low-recruitment conditions, which occurred primarily at high IN–TC scaling values, were retained in the sweep for completeness but should not be interpreted as robustly synchronized network states.

### Sleep spindle detection and statistical analysis

All simulations were conducted with core and matrix Ctx mixing, meaning that core and matrix circuits interacted at the level of the cortex, through connections (mixing/integration) across layers or across areas. Sleep spindles for core and matrix activity were calculated by averaging the neural activity of five core and five matrix neurons at a time, separately from the functionally mixed TC circuit. Mixed spindles were calculated by averaging the combined activity of 10 Ctx neurons (5 matrix and 5 core) from the functionally mixed TC loop. For sleep spindle-like event detection, all averaged activity was bandpass filtered in the 9–13 Hz range (Figure 1E; Figure S8).

We assessed sleep spindle amplitude, duration, and density following the criteria outlined by Gonzalez et al. [61] Spindles were included in the analysis if their amplitude exceeded a set threshold (30% of peak spindle amplitude) and was not within 0.5 s of another detected amplitude [61]. Because the unfiltered signals contained both broad activity changes and faster spindle-like oscillations, detected spindle-like events were identified from bandpass-filtered signal rather than from the full raw trace. Thus, detected events represent intervals where the spindle-band amplitude envelope exceeded threshold, rather than the entire active period visible in the raw signal. Spindle duration was defined as the time difference between two local minima in reference to a detected amplitude. Spindle density was calculated by counting the number of amplitudes within a bandpass-filtered averaged activity array. All spindle metrics were visually cross-validated to ensure accuracy.

Because spindle metrics (amplitude, duration, and density) exhibited non-normal distributions, we performed rank-ordered statistical analysis. Kruskal–Wallis tests were performed to evaluate statistical significance between rodent-like and primate-like spindle metrics (sleep spindle amplitude, duration and density) across all three loop configurations (closed, open, hybrid) (Figures S2–S4).

The 9–13 Hz filter was used for primary spindle event detection, whereas the 10–16 Hz filter was used for power spectral density (PSD)-based spectral quantification to capture the broader spindle/sigma range described in the Introduction. We computed PSD from bandpass-filtered averaged Ctx signals to analyze spectral power in rodent-like and primate-like TC circuits across open, closed, and hybrid loop configurations. For each condition, Ctx neurons were divided into 5 subpopulation blocks. Within each block, activity was averaged across neurons to generate a block-level neural signal. Each block of averaged neuron population activity was then bandpass filtered from 10–16 Hz, after which Fourier transform (FFT) was applied to the filtered signal and power was computed (Figure 7). Mean PSDs were plotted on a logarithmic scale with SEM shading across the 8–20 Hz range to show both average spectral structure and variability across Ctx subpopulations.

Because spindle frequency boundaries vary across species, Ctx regions, and recording techniques, we operationally defined 10–12 Hz as lower and 13–15 Hz as higher spindle/sigma subranges. While exact cutoffs differ across studies, this choice is consistent with prior distinctions between lower-frequency slow spindles and higher-frequency fast spindles [62–67]. To quantify spectral structure, we measured 10–12 Hz power, 13–15 Hz power, the 13–15/10–12 Hz fast/slow ratio, and the most prominent peak frequency within 8–20 Hz. Values were reported as mean ± SEM across Ctx subpopulation blocks. Similarly to the approach of Fernandez and Lüthi [62], our goal was to continuously assess TC spindle activity rather than focus solely on discrete spindle detection. By examining the spectral distribution, we aimed to detect species- and condition-specific differences in content and organization of frequencies within the spindle range, including broad sigma-band shoulders and distributed frequency components. This spectral approach is particularly relevant for comparing rodent-like and primate-like circuits, where differences in spindle expression may reflect species-specific adaptations in TC architecture.

## Results

### Activity in rodent-like and primate-like TC circuit across the parameter search

**Figure 2:**
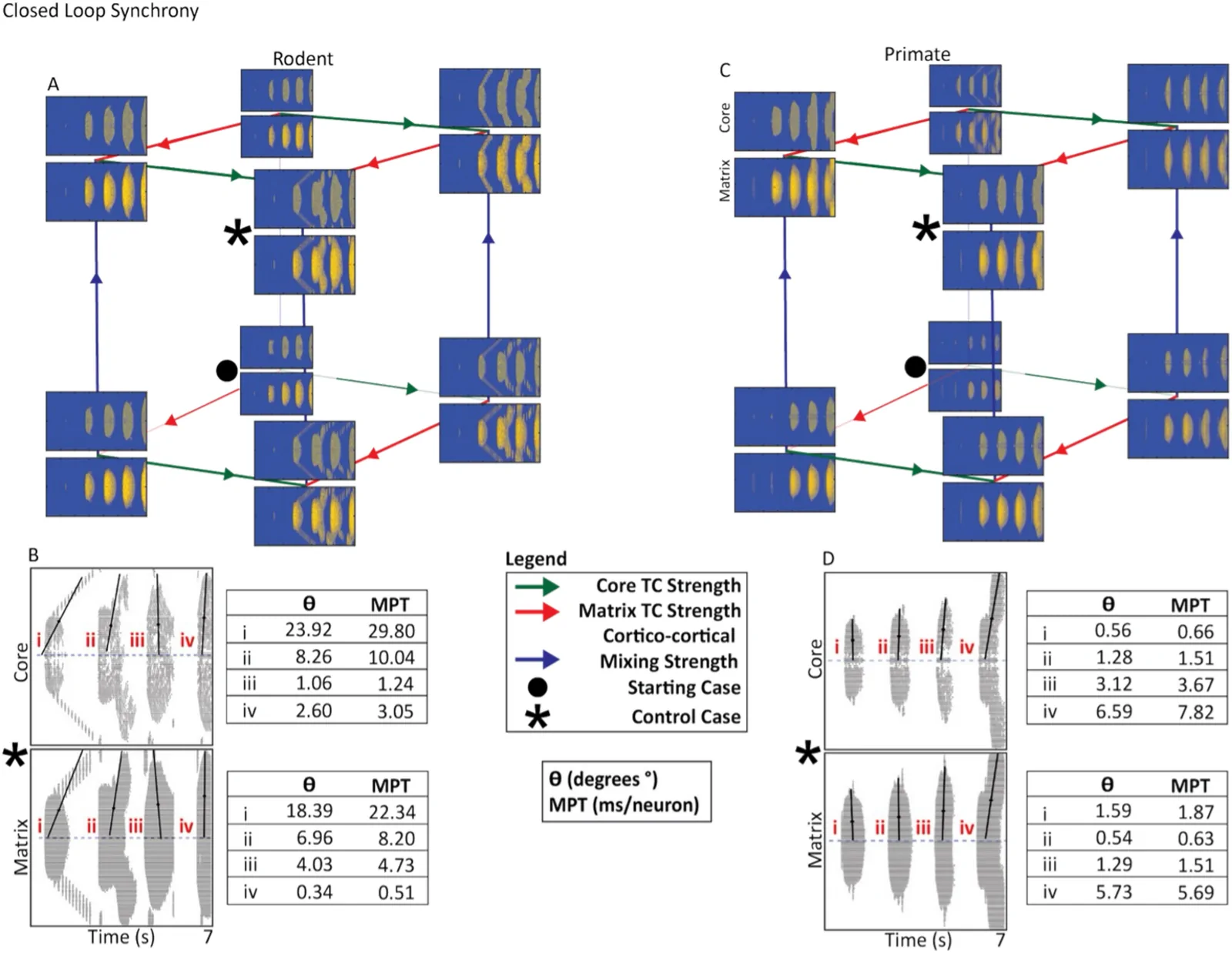
Closed TC loop Ctx synchrony. (A and C) Rodent-like and primate-like cubes present the three-way parameter search of core and matrix TC strength as well as cortico–Ctx mixing strength. The • indicates the starting case, while the * indicates the control case. We assessed changes in synchrony using the control case. Primate-like Ctx activity synchrony and spread was higher than rodent-like Ctx activity. (B and D) Raster plots present control case rodent-like and primate-like Ctx activity from the core and matrix. Each band of activity analyzed is marked with a roman numeral. Deviation-from-vertical angle (θ) and MPT are shown for each activity band. θ (reported in °) reflects burst synchrony based on the orientation of the PC1 eigenvector, while MPT (reported in ms/neuron) was derived from the slope of this eigenvector, representing temporal offset per neuron during burst propagation. Larger θ and MPT values indicate slower, more sequential propagation, whereas smaller values reflect more synchronous activation (see *TC activity synchronization and statistical analysis* section).

**Figure 3:**
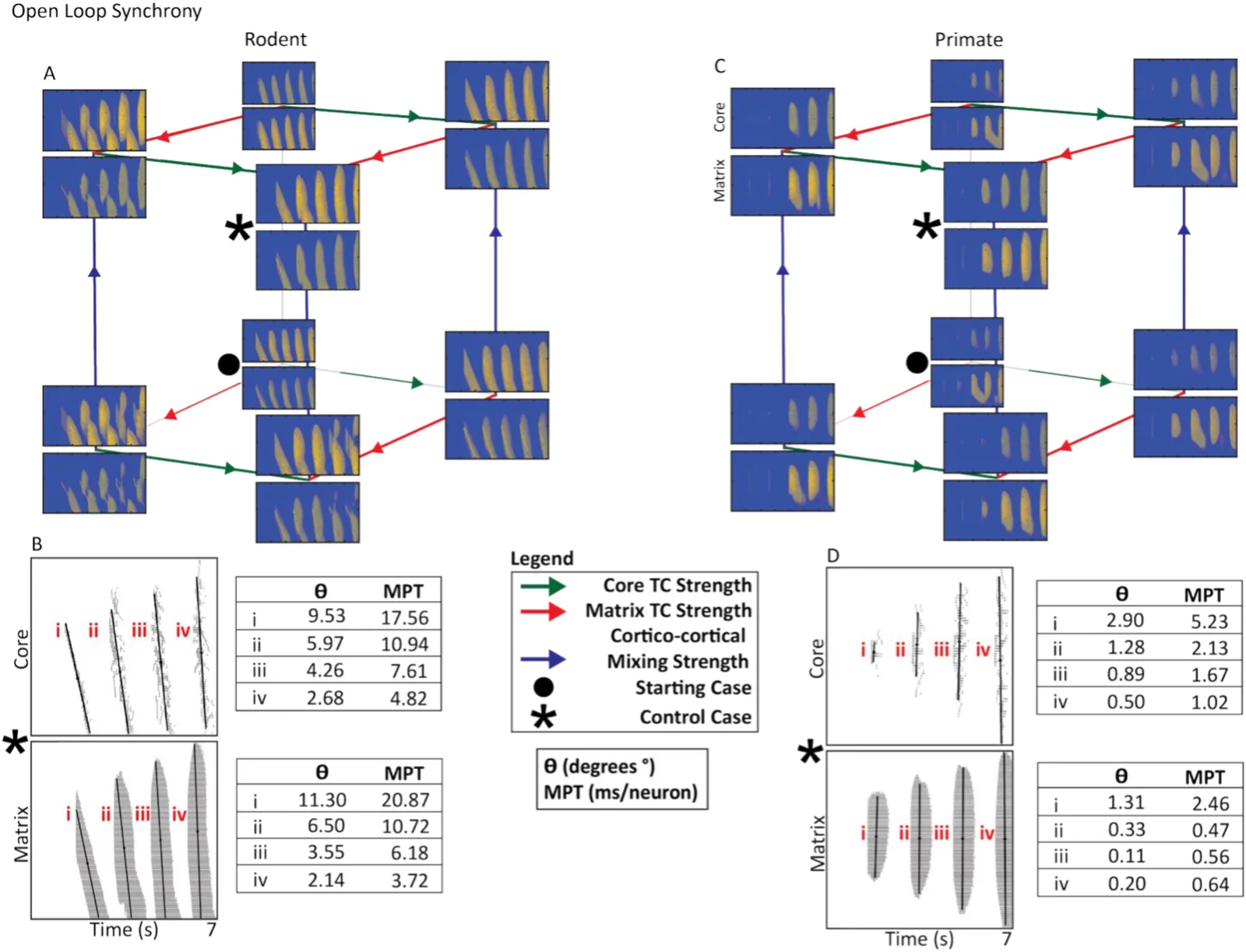
Open TC loop Ctx synchrony. (A and C) Rodent-like and primate-like cubes present the three-way parameter search of core and matrix TC strength as well as cortico–Ctx mixing strength. The • indicates the starting case, while the * indicates the control case. We assessed changes in synchrony using the control case. Primate-like Ctx activity synchrony and spread was higher than rodent-like Ctx activity. Rodent-like Ctx activity showed more prominent traveling-wave structure, consistent with less synchronous recruitment. (B and D) Raster plots present control case rodent-like and primate-like Ctx activity from the core and matrix. Each band of activity analyzed is marked with a roman numeral. Rodent-like activity bands encompassed more neurons than primate-like activity bands. Deviation-from-vertical angle (θ) and MPT are shown for each activity band. θ (reported in °) reflects burst synchrony based on the orientation of the PC1 eigenvector, while MPT (reported in ms/neuron) was derived from the slope of this eigenvector, representing temporal offset per neuron during burst propagation. Larger θ and MPT values indicate slower, more sequential propagation, whereas smaller values reflect more synchronous activation (see *TC activity synchronization and statistical analysis* section*).*

**Figure 4:**
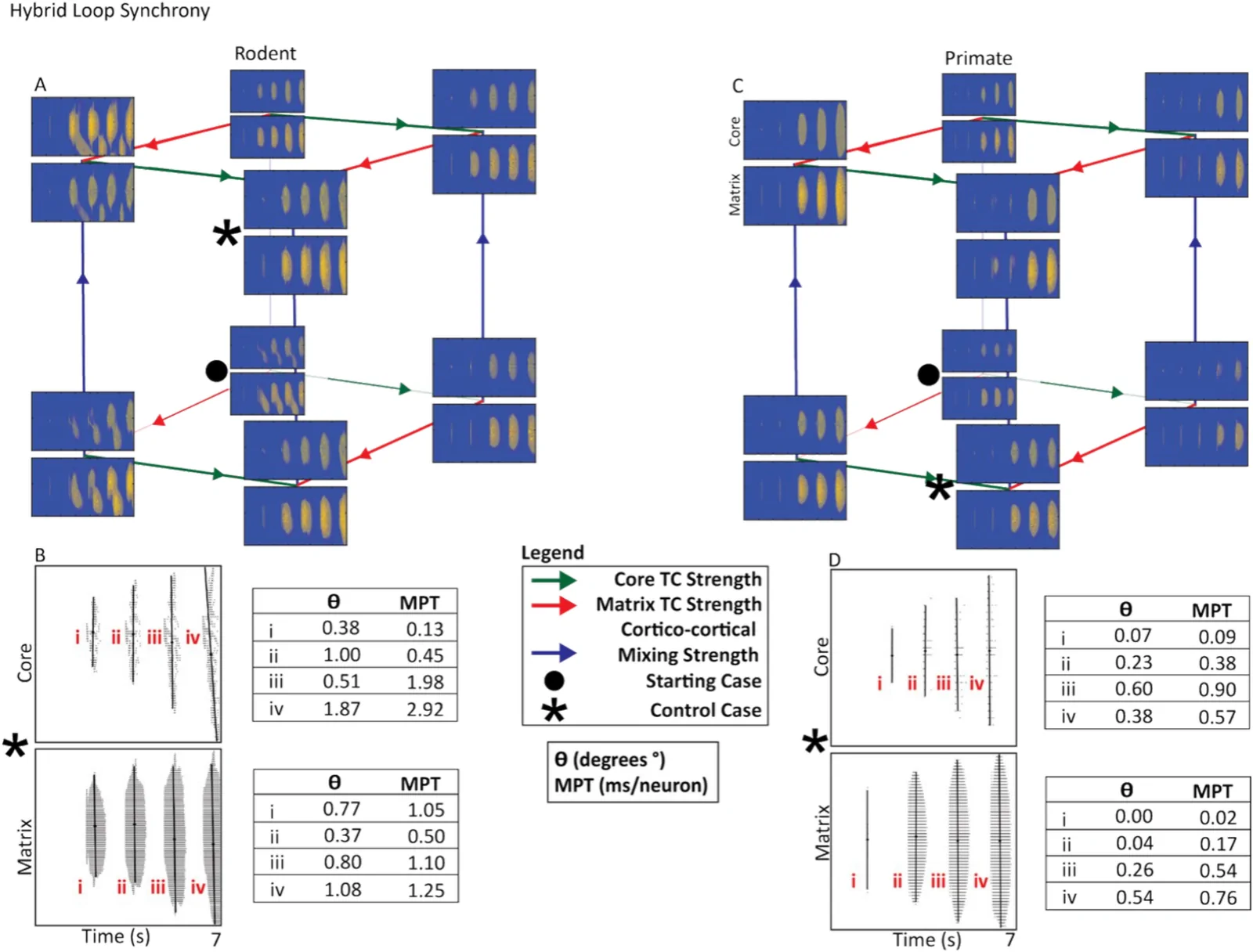
Hybrid TC loop Ctx synchrony. (A and C) Rodent-like and primate-like cubes present the three-way parameter search of core and matrix TC strength as well as cortico–Ctx mixing strength. The • indicates the starting case, while the * indicates the control case. The control case determined in the hybrid loop required lower levels of cortico–Ctx mixing. We assessed changes in synchrony using the control case. Primate-like Ctx activity was more synchronized than rodent-like Ctx activity. (B and D) Raster plots present control case rodent-like and primate-like Ctx activity from the core and matrix. Each band of activity analyzed is marked with a roman numeral. Deviation-from-vertical angle (θ) and MPT are shown for each activity band. θ (reported in °) reflects burst synchrony based on the orientation of the PC1 eigenvector, while MPT (reported in ms/neuron) was derived from the slope of this eigenvector, representing temporal offset per neuron during burst propagation. Larger θ and MPT values indicate slower, more sequential propagation, whereas smaller values reflect more synchronous activation (see *TC activity synchronization and statistical analysis* section)*.*

In both species-inspired models, the closed loop produced symmetrical, vertically aligned activity bands (Figure 2). In contrast, the open and, to a lesser extent, hybrid loops generated asymmetrical bands characteristic of traveling waves (Figures 3–4). Traveling waves were most prominent in the rodent-like model but were also observed under certain parameter settings in the primate-like model.

In both rodent-like and primate-like closed loops, the spread of Ctx activity increased as a function of core and matrix TC strength. In the rodent-like model, increasing core TC strength altered the synchrony of Ctx activity, producing more tilted activity bands and traveling-wave structure, whereas the primate-like model was largely unaffected. Additionally, increased cortico–Ctx mixing amplified the spread of Ctx activity in both rodent-like and primate-like closed loops (Figure 2A and C). Differences in the effect of core TC strength on synchrony led to drastically different control cases in the rodent-like and primate-like models.

The rodent-like open loop generated traveling waves, regardless of the values of core TC strength, matrix TC strength, or cortico–Ctx mixing. The primate-like open loop, while still capable of generating asymmetrical bands in some parameterizations, often produced more organized, less prominent traveling waves, partially resembling the closed loop geometry (Figure 3C •). Across the parameter search, neither model showed major changes in the spread of activity as a function of increased core or matrix TC strength. In the rodent-like open loop, increasing matrix TC strength caused separation in the traveling waves (Figure 3A). In the primate-like open loop, increased matrix, and to a lesser extent, core TC strength, reduced the prominence of traveling waves (Figure 3D).

In the rodent-like hybrid loop, increasing core TC strength resulted in more synchronized and cohesive activity bands (Figure 4A and C). In contrast, increasing matrix TC strength heightened traveling waves and separation. For the primate-like hybrid loop, increasing cortico–Ctx strength caused an increase in the spread of Ctx activity. However, even at the most extreme parameters, primate-like activity bands remained substantially more synchronous than those in the rodent-like model (Figure 4B and D).

Before presenting the key findings from each loop configuration, we wanted to note general trends that were present in all three versions of the TC loop and in both species-inspired models. Matrix Ctx activity tended to synchronize more than the core Ctx activity. Across the parameter search for both species, matrix-derived Ctx activity appeared more diffuse than core activity, although this difference was far more pronounced in the rodent-like model. Figures S5 and S6 show representative spatiotemporal rasters from additional neuronal populations, including TRN, TC, and interneuron populations, where present. Corresponding burst-window activation summaries for these examples are provided in Table S7.

### Spatiotemporal structure of TC activity across loop configurations

Across simulations, TC dynamics showed configuration- and species-dependent differences in the temporal organization of activity bands. PCA-derived eigenvector angle and MPT revealed consistent patterns in the temporal structure of TC activity bands. The rodent-like network systematically produced activity bands with greater temporal dispersion, whereas the primate-like networks generated more aligned activity. These species differences were evident across open, closed, and hybrid loop configurations and were expressed similarly across core and matrix pathways.

### PCA- and MPT-based activity band structure and dimensionality

Activity band angles in the rodent-like model tended to deviate more from the vertical, indicating that rodent-like activity bands exhibited stronger temporal shift and a greater degree of sequential activation across the neuronal population. Correspondingly, rodent-like MPT values were consistently higher, reflecting a larger elapsed time per neuron along the activity band. Together, these metrics showed that rodent-like TC circuits tended to exhibit activity that travels gradually across neighboring neurons rather than occur simultaneously. Primate-like loop configurations showed the opposite pattern, with near vertical PCA orientations, indicating minimal temporal tilt and a high degree of activity concurrence within each band. Primate-like MPT values were accordingly small, indicating minimal temporal spread across neurons. These results may suggest that primate-like TC loops support more synchronized population events.

Loop configurations further regulated these species-specific tendencies. Open and closed-core circuits amplified the differences, exhibiting the strongest propagation patterns in rodent-like loops and the most synchronous activity in primate-like loops. In contrast, hybrid configurations were highly synchronous in both species-inspired models. In these hybrid loops, θ values were consistently near 0°, and MPT values were also small, indicating that the mixed connectivity pattern was associated with more synchronized firing across both species-inspired architectures. Across all analyses, PCA and MPT showed consistent patterns: rodent-like loops tended to exhibit more sequential firing, whereas primate-like loops showed more synchronized activity.

### Activity synchrony of the closed TRN–TC loop

The primate-like closed loop consistently displayed higher levels of synchrony, regardless of cortico–Ctx or core and matrix TC connectivity changes, unlike the rodent-like closed loop (Figure 2A and C). While both species-inspired models exhibited symmetrical activity bands in the closed architecture, rodent-like synchrony level was more sensitive to parameter variation: increases in core TC strength altered the spatiotemporal pattern of rodent-like Ctx activity, whereas primate-like activity retained comparatively stable synchrony level. These differences were also reflected in the PCA and MPT analyses (Figure 2B and D).

Rodent-like PCA angles deviated more prominently from vertical, notably in the core condition where θ reached 23.92°, corresponding to an MPT of 9.59 ms/neuron, indicating strong temporal tilt and sequential propagation. In contrast, primate-like closed-core θ values remained closer to 0° (0.56°–6.59°) with smaller MPT values (1.38–3.98 ms/neuron), reflecting more synchronous activation. Similar patterns emerged in the matrix pathway: rodent-like deviations ranged 2.65°–6.22° (MPT: 0.45–4.54 ms/neuron), whereas primate-like deviations remained tighter (1.67°–3.13°) with lower MPTs (0.94–3.28 ms/neuron).

Additionally, we observed species-specific differences in how synchrony evolved across the activity bands over the time course of the simulation. In primates-like model, core and matrix Ctx bands began in a highly synchronous configuration and remained vertically aligned throughout the progression of the simulation, with PCA orientations remaining close to vertical (corresponding to θ values near 0°) and consistently minimal MPT values (Figure 2C and D). In contrast, the rodent-like model began in a less synchronized state, with tilted activity bands in the early stages of the simulation, which then became more vertically organized as the simulation progressed (Figure 2A and B). This gradual synchronization of rodent-like activity bands was reflected in PCA deviations that moved closer to 0° and in decreasing MPT values over the course of the simulation. Thus, while primate-like closed-loop synchrony was stable and appeared inherent, rodent-like synchrony likely had to develop over time.

### Activity synchrony of the open TRN–TC loop

The rodent-like open loop was dominated by traveling waves regardless of the cortico–Ctx, core or matrix TC connectivity (Figure 3A). PCA captured this pattern of sequential propagation: rodent-like activity band orientations showed clear deviations from vertical (2.14°–11.30°) along with elevated MPT values (3.72–20.87 ms/neuron) (Figure 3B). The primate-like open loop also exhibited traveling-wave activity, but these waves were less pronounced than those observed in the rodent-like open loop (Figure 3C). Accordingly, primate-like activity band deviations remained small (0.87°–2.90°) and produced substantially lower MPT values (0.47–5.23 ms/neuron) than in the rodent-like model, indicating higher synchrony. In the primate-like open loop, increased core or matrix TC strength reduced the prominence of traveling waves, resulting in a more synchronous activity pattern (Figure 3D). In contrast, in the rodent-like open loop, increases in matrix TC strength caused greater separation among traveling waves (Figure 3A). As such, PCA and MPT jointly indicated higher synchrony in the primate-like compared to the rodent-like model.

### IN–TC inhibitory connectivity modulates synchrony and propagation

**Figure 5:**
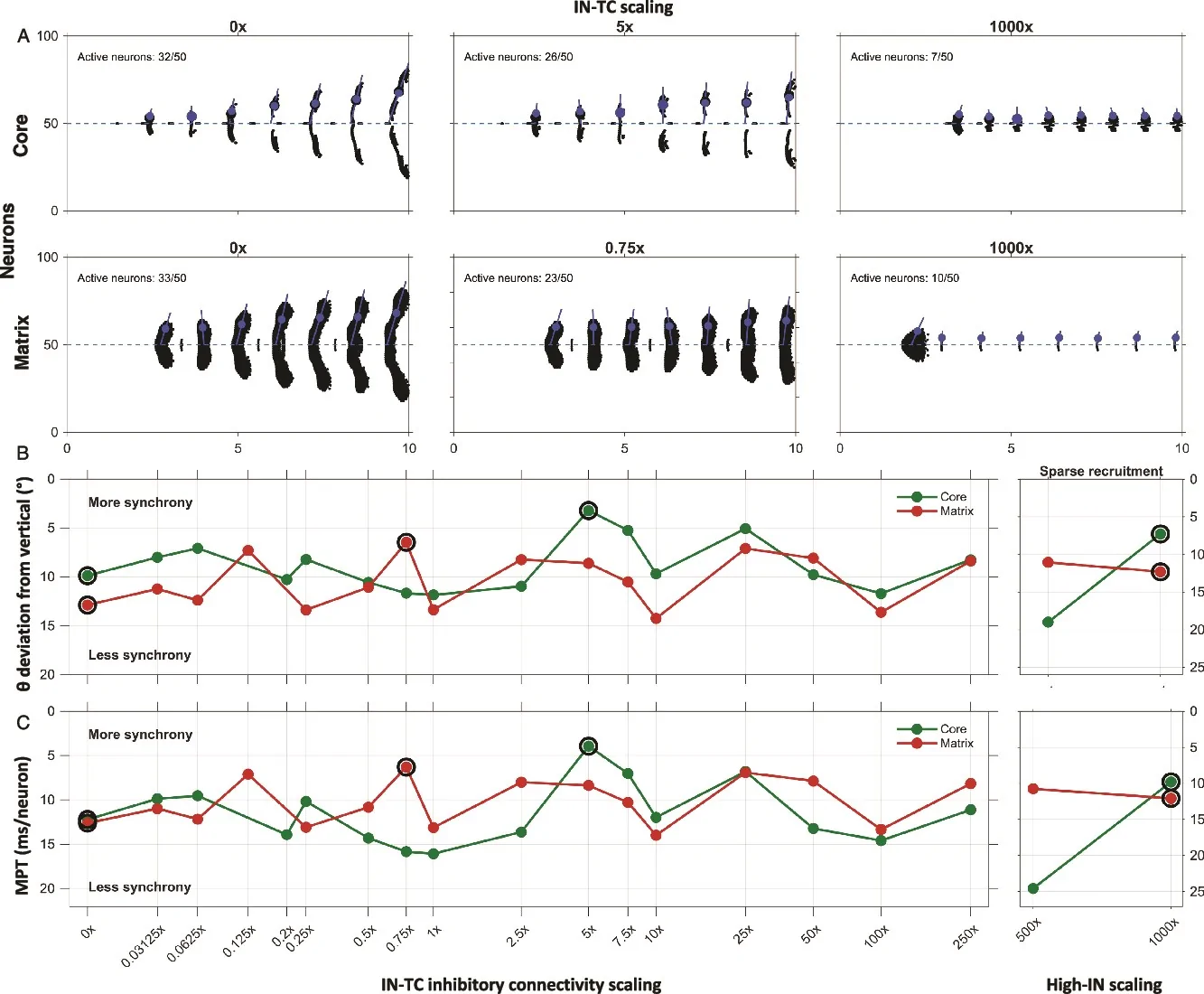
IN–TC inhibitory connectivity scaling modulates primate-like synchrony and propagation. IN–TC inhibitory connectivity strength was scaled in the primate-like model. (A) Representative core and matrix rastergrams at selected IN–TC scaling values. PCA-derived orientation vectors, shown as lines extending from centroid markers, indicate the orientation of the activity band; dashed horizontal lines mark the population center, and active-neuron counts indicate recruitment. (B) PCA-derived absolute deviation from vertical, θ (°), across the scaling sweep. C. MPT (ms/neuron), across the scaling sweep. The *y*-axes are inverted, with 0 at the top, because lower θ and lower MPT indicate greater synchrony. Thus, upward shifts indicate more synchronous activity, while downward shifts indicate less synchronous or more propagating activity. The *x*-axis is split so that very high IN–TC scaling values are shown separately on the right. These high-IN conditions produced sparse recruitment and are included for completeness, but they should not be interpreted as robust synchronized network states.

To directly test whether thalamic interneuron-mediated inhibition contributes to synchrony and propagation, we performed an additional parameter sweep in the primate-like model in which the inhibitory connectivity strength from thalamic IN onto TC relay neurons was scaled from 0 to 1000 times its baseline value with logarithmic steps (Figure 5). A scaling factor of 1 corresponded to the original primate-like model parameterization, whereas a scaling factor of 0 removed the GABAergic inhibitory current from thalamic IN onto TC relay neurons while leaving the rest of the primate-like circuit unchanged. Thus, the 0 scaling factor condition isolates the absence of local thalamic interneuron inhibition, which is a rodent-like architectural feature in our simplified model.

Across the sweep, we quantified PCA-derived deviation from vertical alignment (θ), MPT per neuron, and active neuron recruitment, showing that varying IN–TC inhibitory connectivity strength altered both synchrony and population recruitment. Initially, starting from the scaling factor of 0, increasing inhibitory connectivity strength shifted activity toward stronger synchrony, as indicated by more vertically aligned activity bands, corresponding reductions in PCA-derived deviation from vertical alignment, θ, and lower MPT values (Figure 5A–C). However, this relationship was not monotonic across the full parameter sweep. Synchrony was strongest at selected intermediate scaling values, including local minima in MPT, indicating that increasing inhibitory connectivity strength can shift activity toward synchrony within part of the tested parameter space.

Further increases in inhibitory connectivity strength did not produce a consistent progression toward stronger synchrony. Instead, synchrony and propagation varied across the sweep, suggesting that the effect of thalamic interneuron-mediated inhibition depends on the broader excitatory–inhibitory balance of the model (Figure 5B and C). At the highest inhibitory scaling values, activity became restricted to fewer neurons, particularly in the core compartment (Figure 5A). These high-scaling conditions therefore suggest reduced population recruitment rather than a simple increase in network synchrony. Together, these results indicate that thalamic interneuron-mediated inhibition can support more synchronous activity within selected parts of the tested parameter space, but its effect is not a monotonic consequence of increasing inhibitory connectivity strength.

### Synchrony across circuit nodes in the hybrid TRN–TC loop

Hybrid-loop architecture produced the most synchronized patterns of activity bands for both species. Rodent-like hybrid activity was very sensitive to the parameter changes in connectivity, as increasing core TC strength or cortico–Ctx mixing produced more vertically aligned activity bands (Figure 4A), reflecting a shift toward synchrony. This was supported by PCA-derived deviation-from-vertical θ values that approached zero in both the core pathway (0.38°–1.87°) and in matrix pathway (0.77°–1.08°), with correspondingly small MPT values (Figure 4B), indicating reduced temporal dispersion across neurons. In contrast, increasing matrix TC strength had the opposite effect as activity bands became more asymmetric and more widely separated, producing traveling waves (Figure 4A). Primate-like hybrid loop remained more synchronous overall, even as core TC strength, matrix TC strength, and cortico–Ctx mixing were varied (Figure 4C). Activity bands stayed nearly vertical, with PCA-derived eigenvector deviations consistently near zero (0.07°–0.60° in the core pathway; 0.00°–0.54° in the matrix pathway) and extremely small MPT values (0.02–0.90 ms/neuron) (Figure 4D), indicating near-synchronous population activation.

Together, the hybrid loop results reveal that rodent-like loops are capable of producing both synchronous and asynchronous activity, depending on whether core TC strength or cortico–Ctx mixing dominates the circuit. By contrast, primate-like loops remain consistently synchronous.

### Sleep spindle metrics

**Figure 6:**
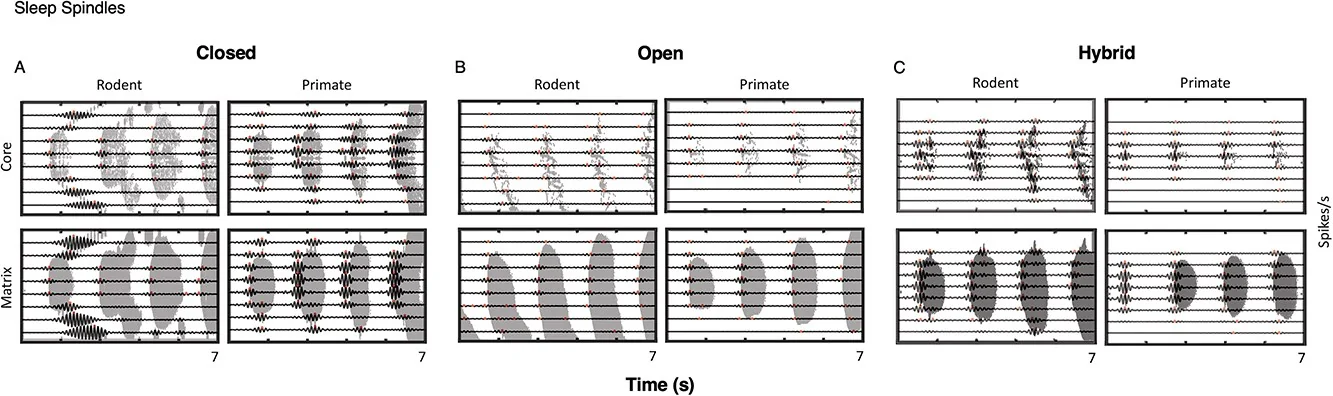
Rodent-like and primate-like TC loop spindles across loop configurations. Core and matrix sleep spindle examples in rodent-like and primate-like models. In each panel, representative spindle traces are shown as waveforms, detected spindle events are marked with red point markers, and shading indicates the associated activity periods. Raster activity bands reflect broader network activity and are not equivalent to individual detected spindle-like events, which were quantified separately using the filtered-signal envelope. (A) Closed loop. Rodent-like spindles appeared more variable and broader in shape in both core and matrix pathways, with no strong separation between the two. Primate-like spindles showed more uniform structure across core and matrix, with clearer boundaries and more consistent timing across events. (B) Open loop. Rodent-like core and matrix spindles showed greater spatiotemporal variability, with spindle shapes that were less uniform and lower in amplitude, whereas primate-like spindles, both core and matrix, appeared to be more stable in shape and more regularly spaced. (C) Hybrid loop. Hybrid loop simulations showed the same overall pattern: there was no strong core–matrix distinction in either species, but rodent-like spindles were more variable overall, whereas primate-like spindles maintained a more uniform structure across events. Across all loop configurations, the rodent-like model displayed greater spindle spatiotemporal variability, while the primate-like model showed more coherent spindles.

We assessed changes in sleep spindle metrics (amplitude, duration and density) between rodent-like and primate-like as a function of their reticulo–thalamic (closed, open, or hybrid loop) connectivity. Representative examples of core, matrix, and mixed spindle signals are shown in Figure 6, while quantitative summaries of spindle amplitude, duration, and density are provided in Figures S2–S4.

In the closed loop, rodent-like core (*p* = 5.95*10^-6^, α = 0.05) and mixed (*p* = 1.54*10^-5^, α = 0.05) sleep spindle amplitudes were significantly higher than primate-like core and mixed spindle amplitudes (Figure S2B). In the open loop, primate-like matrix sleep spindle duration was significantly higher than rodent-like matrix spindle duration (*p* = 7.4*10^-3^, α = 0.05), whereas rodent-like spindle density was significantly higher for all three spindle types (Core: *p* = 3.35*10^-2^, α = 0.05, Matrix: *p* = 9.94*10^-5^, α = 0.05, Mixed: *p* = 1.32*10^-2^, α = 0.05) (Figure S3B). In the hybrid loop, rodent-like core and mixed sleep spindle amplitude was significantly higher than primate-like core and mixed sleep spindle amplitude (Core: *p* = 9.57*10^-7^, α = 0.05 and Mixed: *p* = 2.00*10^-4^, α = 0.05), and rodent-like mixed spindle density was also significantly higher than primate-like mixed spindle density (*p* = 1.23*10^-2^, α = 0.05) (Figure S4B).

We also examined the distributions of spindle amplitude, duration, and density across loop configurations (Figures S2C and D, S3C and D, and  S4C and D). Amplitude distributions differed across species and loop conditions, with broader primate-like amplitude variability in some cases and greater loop-dependent variation in rodent-like amplitudes. Spindle duration distributions were bimodal in both rodent-like and primate-like models, with events clustering around shorter (~1 s) and longer (~3 s) durations. By contrast, density distributions did not show clear species-dependent differences in variability across loop configurations, despite the significant mean differences noted above.

We also assessed sleep spindles across rodent-like and primate-like models as a function of their reticulo–thalamic (closed, open or hybrid loop) connectivity. Figure 6A, B, and  C, present examples of core and matrix sleep spindles generated by the three versions of the rodent-like and primate-like model. Across all three loop architectures, clear model-dependent differences were visible in the structure and consistency of spindle activity. In the rodent-like model, both core and matrix spindles appeared more spatiotemporally heterogeneous. The shaded activity bands varied noticeably in width and shape, and the number of neurons participating in each spindle shifted across events. In addition, the rodent-like model showed more pronounced traveling-wave structure and less uniform spacing of the activity bands from which spindle events were extracted.

In the primate-like model, core and matrix spindles appeared more consistent and temporally aligned. The spindles were more regular in shape and had clearer boundaries. Successive spindles had more regular duration and spacing, and the overall pattern of activity appeared more stable across loop types. Primate-like core and matrix circuits displayed this increased regularity relative to the rodent-like model. Altogether, the spindle patterns indicate greater spatiotemporal variability in the rodent-like model, while the primate-like model shows more coherent and consistently structured spindles across core and matrix of all three TC loop configurations.

### PSD analysis of TC activity

**Figure 7:**
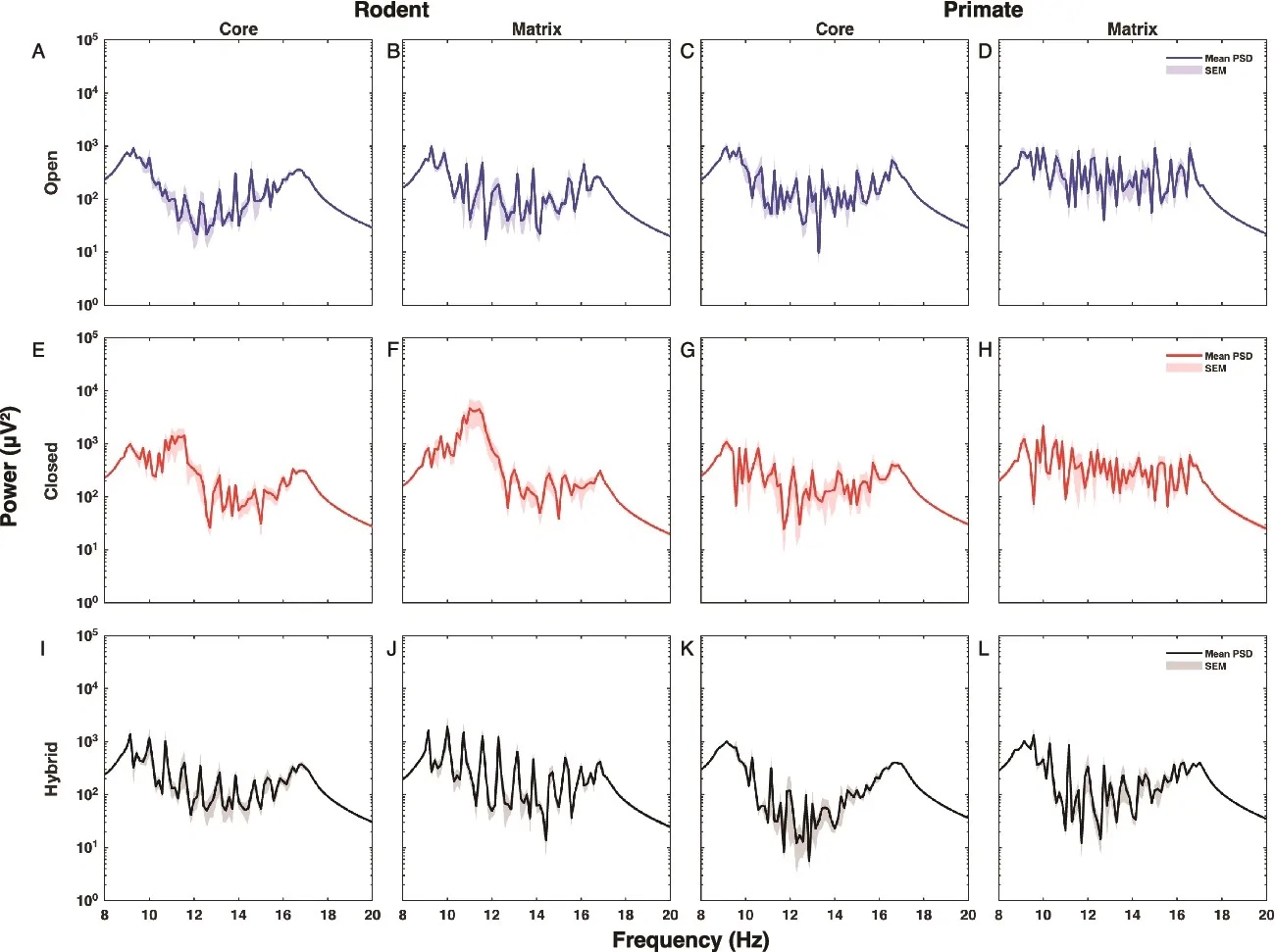
Spectral power across rodent-like and primate-like TC loop configurations. PSDs of averaged simulated signals are shown for TC core (left) and matrix (right) pathways. (A–D) Open-loop configurations. (E–H) Closed-loop configurations. (I–L) Hybrid-loop configurations. Rodent-like models are shown in A–B, E–F, and I–J; and primate-like models are shown in C–D, G–H, and K–L. Signals were bandpass filtered from 10–16 Hz before FFT-based PSD calculation. Power outside the 10–16 Hz passband is shown for visualization of adjacent frequencies but is expected to be attenuated by the bandpass filter; quantitative interpretation focused on the 10–12 Hz and 13–15 Hz subranges. PSDs were plotted on a logarithmic scale from 8 to 20 Hz, covering the spindle frequency range (~10–16 Hz) and adjacent bands. Shaded regions indicate SEM across Ctx subpopulations. Open loop configuration showed relatively broad spectral profiles without strongly isolated peaks. Closed loops displayed enhanced low-frequency spindle-band structure (~10–12), particularly in the rodent-like matrix pathway. Hybrid loops showed activity distributed across the spindle range, often with multiple local peaks. Overall, rodent-like models showed relatively greater contribution from the lower spindle frequencies in the closed-loop conditions, whereas primate-like models exhibited broader spindle-band distributions across open and hybrid loop configurations.

PSD analysis showed that both rodent-like and primate-like models exhibited sustained activity in the spindle range across loop configurations (Figure 7). To quantify spectral structure beyond visual inspection, we measured lower spindle-band range (10–12 Hz) power, higher spindle-band range (13–15 Hz) power, fast/slow ratio, and peak frequency for each species, pathway, and loop configuration (Table S8).

In the open loop configuration (Figure 7A–D), both species-inspired models showed measurable 10–12 Hz and 13–15 Hz band power in core and matrix pathways. In the core pathway, primate-like circuits showed slightly higher 10–12 Hz and 13–15 Hz power than rodent-like circuits (10–12 Hz: 189.18 ± 77.24 vs. 143.46 ± 39.12 μV [2]; 13–15 Hz: 131.35 ± 48.93 vs. 105.77 ± 38.74 μV [2]; Figure 7A and C). This difference was more pronounced in the matrix pathway, where primate-like circuits showed higher 10–12 Hz and 13–15 Hz power than rodent-like circuits (10–12 Hz: 366.31 ± 136.08 vs. 185.29 ± 55.90 μV [2]; 13–15 Hz: 286.62 ± 134.16 vs. 101.76 ± 32.96 μV [2]; Figure 7B and D). Peak frequencies remained in the sigma/spindle range in both species, ranging from 11.3 to 13.3 Hz.

Closed loop configurations (Figure 7E–H) exhibited a stronger shift toward lower spindle-band power. In rodent-like circuits (Figure 7E and F), peak frequencies shifted lower to 11.3 ± 0.2 Hz in core and 10.9 ± 0.2 Hz in matrix, with 10–12 Hz power clearly exceeding 13–15 Hz power in both pathways. This effect was strongest in rodent closed matrix, where 10–12 Hz power reached 2379.66 ± 1038.37 μV [2] compared with 147.33 ± 30.31 μV [2] in the 13–15 Hz range (Figure 7F). Primate-like circuits (Figure 7G and H) also showed lower frequency peaks at 10.5 ± 0.1 Hz in core and 10.6 ± 0.1 Hz in matrix. However, primate-like circuits were less dominated by the 10–12 Hz range than rodent-like circuits, with higher fast/slow ratios in both core and matrix pathways.

In the hybrid loop (Figure 7I–L), we observed sustained spindle-band activity in both species. Rodent-like hybrid circuits (Figure 7I and J) peaked at 11.5 ± 1.0 Hz in core and 10.8 ± 0.4 Hz in matrix, whereas primate-like hybrid circuits (Figure 7K and L) peaked at 10.2 ± 0.1 Hz in core and 11.2 ± 0.8 Hz in matrix. Rodent-like hybrid pathways showed higher absolute power than primate-like hybrid pathways in both the 10–12 Hz and 13–15 Hz ranges. For example, rodent hybrid matrix showed 489.21 ± 195.06 μV [2] in the 10–12 Hz range and 166.12 ± 65.19 μV [2] in the 13–15 Hz range, whereas primate hybrid matrix showed 249.23 ± 107.62 μV [2] and 128.25 ± 51.25 μV [2], respectively (Figure 7J and L). However, primate-like hybrid circuits had relatively higher fast/slow ratios, indicating that they were less dominated by lower spindle-band power than rodent-like hybrid circuits.

Overall, these results indicate that spindle-band activity is present across all conditions, but its frequency distribution depends on both species-inspired model architecture and loop architecture. Rodent-like circuits displayed stronger lower-frequency spindle-band structure in closed and hybrid loop configurations, especially in the closed matrix pathway. Primate-like circuits were generally less dominated by the lower spindle range, as shown by higher 13–15/10–12 Hz power ratios than the matched rodent-like condition across all loop and pathway comparisons.

## Discussion

### Activity in rodent-like vs. primate-like TC model circuits

This study explored how several critical TC circuit organizational principles can affect TC network dynamics, in particular sleep spindle activity characteristics, with possible implications for future experimental work and computational modeling. Our findings revealed species-specific patterns of activity between distinct core and matrix TC circuits, each including three different thalamoreticular loop configurations (open, closed, hybrid).

This study used reduced, species-inspired TC network models to explore how selected architectural features that are distinct across species, affect synchrony, propagation, and spindle-like activity. These features include specialized patterns of core versus matrix connectivity organization, thalamic interneuron inclusion, and distinct TRN–thalamic loop configurations. Our results should therefore be interpreted as mechanistic comparisons between two model variants rather than comprehensive reconstruction of rodent and primate network functioning. Within that scope, the primate-like model showed more stable synchrony across the tested parameters, whereas the rodent-like model was more sensitive to changes in TC conductance and more likely to display propagating or traveling-wave-like activity. These differences suggest that the modeled organizational features are sufficient to influence the balance between temporal stability and propagation in TC circuits.

The simulated TC circuit activity of primates and rodents showed key differences, because of the presence of IN. Differences in interneuron involvement across species contribute to variations in circuit stability and responsiveness [68]. Primates exhibit a more differentiated core and matrix structure in thalamic projections, with prominent interneuron-mediated inhibition, enhancing feedback loops and regulating spiking dynamics [29, 48, 69–71]. Rodent models, on the other hand, provide valuable insights into closed-loop configurations, where their circuitry demonstrates heightened sensitivity to changes in TC strength [72]. Lacking significant interneuron activity (except in the LGN and few other first-order nuclei), rodent circuits rely more on direct TC excitation and inhibition, making them more sensitive to changes in TC connectivity [71, 73–76]. This sensitivity makes rodents an excellent model organism for studying fundamental principles of circuit plasticity and parameter-driven variations in synchrony. Additionally, the lack of significant interneuronal activity in rodent circuits allows for a more targeted examination of core network dynamics. These differences highlight the need for computational models to incorporate the distinct roles of IN and their dynamics to accurately simulate primate neurophysiology.

### Synchrony and loop configurations

In the closed-loop configurations, primate-like circuits consistently demonstrated higher synchrony, suggesting greater stability and resilience, possibly due to their more complex Ctx mixing and connectivity patterns [77, 78]. By contrast, the rodent-like closed loop exhibited reduced synchrony and greater sensitivity to changes in core TC strength. These findings emphasize the difference in roles between core and matrix projections in species with different Ctx architectures.

The open loop highlighted significant differences in how rodent-like and primate-like TC circuits handle traveling waves. Rodent-like models exhibited persistent traveling waves across parameter variations, reflecting a greater tendency toward propagating activity in this loop configuration. Meanwhile, primate-like models demonstrated reduced traveling wave magnitude, reflecting more localized and efficient neural processing [78].

The hybrid loop exhibited pronounced differences in synchrony dynamics. The rodent-like model showed increased asymmetry and more pronounced traveling wave separations with increasing matrix strength and with greater recruitment of neurons. In contrast, the primate-like model demonstrated broader activity spread across Ctx regions with increases in core, matrix, and cortico–Ctx strength, without the pronounced asymmetry observed in rodents. One possible explanation for the increased synchrony observed in the hybrid configuration is that combining open and closed motifs increases functional coupling across the loop relative to either extreme architecture alone. In the rodent-like model, this mixed organization may partially stabilize propagation and reduce traveling wave tendencies, whereas the addition of inhibitory structure (IN) in the primate-like model may already diminish propagation. Because anatomical evidence suggests that purely open or purely closed thalamoreticular organization is unlikely to capture the full range of in vivo circuitry, the hybrid configuration may better approximate biologically plausible coordination dynamics. We interpret this as a mechanistic hypothesis generated by the model rather than as a directly demonstrated causal result.

In the present study we performed an exploratory IN–TC inhibitory-connectivity sweep in the primate-like core–matrix closed-loop model. Future work should extend this analysis across additional loop configurations, pathways, and inhibitory parameter regimes to determine how robust the effects of interneuron-mediated inhibition on synchrony and propagation remain across the broader model space. While these results suggest that IN–TC inhibitory connectivity can affect synchrony within the tested range, its effects on spindle occurrence, density, and duration remain an important direction for future work.

The spatiotemporal pattern of activity in the rodent-like model was more sensitive to changes in TC connectivity and exhibited greater variability across loop configurations compared to the primate-like model. This variability reflects the heightened sensitivity of rodent-like circuits to parameter changes, possibly due to their simpler loop architecture and fewer degrees of freedom compared to primates [73, 76, 79]. Parameter changes refer to adjustments in TC connectivity, including the strength of core and matrix projections and the degree of cortico–Ctx mixing implemented in the model. While this sensitivity makes rodents an excellent model for studying fundamental mechanisms of network plasticity and parameter-driven dynamics, the richer TC loops observed in primates, with more degrees of freedom, provide greater stability and adaptability under varying conditions.

### IN–TC inhibitory connectivity may influence TC synchrony

The IN–TC inhibitory-connectivity sweep suggests that thalamic IN may contribute to synchrony in the primate-like model. In the absence of inhibition from thalamic IN onto TC relay neurons, activity was less synchronous. Adding IN–TC inhibitory connectivity initially shifted the model toward more synchronous recruitment, as indicated by lower MPT values and more vertically aligned activity bands. These results suggest that thalamic interneuron-mediated inhibition may help support synchronized activity in the primate-like circuit. This interpretation is consistent with recent evidence showing that the type and strength of thalamic interneuron-mediated inhibition can alter TC relay neuron input selectivity [80].

The effect, however, was not monotonic. Increasing inhibitory connectivity beyond the initial zero value did not produce a uniform shift toward stronger synchrony. Instead, synchrony and MPT varied across the tested parameter space, suggesting that the influence of thalamic interneuron-mediated inhibition depends on the surrounding circuit state, including the balance between inhibitory drive, excitatory recruitment, and population-level participation. The high-inhibition conditions further illustrate this point. At the largest scaling values, activity became restricted to a smaller subset of neurons. Although some activity bands in these conditions appeared highly vertical, this apparent synchrony occurred in the context of reduced population recruitment. These conditions therefore should not be interpreted as evidence that stronger inhibition necessarily improves network synchrony. Rather, they indicate that excessive inhibitory connectivity can limit recruitment while preserving or exaggerating vertical alignment among the remaining active neurons.

Overall, these findings argue against a simple model in which stronger IN–TC inhibition uniformly increases synchrony. Instead, thalamic interneuron-mediated inhibition appears to shape synchrony and recruitment jointly. Within part of the tested parameter space, adding IN–TC inhibition can promote more synchronous recruitment, but at higher scaling values the same projection can restrict the number of participating neurons. Thus, IN–TC inhibitory connectivity should be interpreted as one contributor to primate-like TC synchrony, whose effect depends on the broader excitatory–inhibitory structure of the circuit.

### Synchrony and propagation signatures reveal divergent TC dynamics across species

The PCA and MPT analyses add a mechanistic interpretation to the qualitative differences observed across loop configurations. In primates, activity band PCA orientations remained near vertical, corresponding to θ values near 0° across closed, open, and hybrid architectures, and MPT values remained consistently low, demonstrating that primate-like TC activity bands exhibit highly synchronous neuronal recruitment. Primate-like synchrony remained stable even when direct TRN feedback was removed (open loop) and connectivity parameter variations were pushed toward extreme values, highlighting the stability of primate-like synchrony. Rodent-like circuits showed a different pattern. In multiple configurations, especially the closed loop and the open loop configurations, activity band angles deviated strongly from vertical, with θ values reaching 23.92° in the closed loop and 11.30° in the open loop, and elevated MPT values up to 29.80 ms/neuron. These metrics reflect traveling-wave–like propagation and a substantial temporal spread across neurons, even when the corresponding primate-like condition remained synchronous. Only under specific connectivity parameter combinations, such as reduced core TC strength in the closed loop or increased core TC strength and higher cortico–Ctx mixing in the hybrid loop, did rodent-like loop configurations approach the more synchronous patterns typically seen in primates. Even so, rodent-like synchrony remained less stable. Likewise, primate-like loops, while generally highly synchronous, displayed some desynchronization in cases where core TC strength or matrix TC strength was reduced within the closed and hybrid loops. Together, these results suggest that primate-like loops tend to remain synchronous across most configurations, while rodent-like loops showed a broader range of temporal patterns and reached synchrony only under specific connectivity conditions. In this context, the apparent stability of synchrony in primate-like circuits should not be interpreted as reduced circuit flexibility, but rather as preservation of synchronous population activity across a wide range of connectivity parameters. Importantly, synchrony represents only one dimension of network dynamics and does not fully capture functional flexibility. While rodent-like circuits exhibited flexibility through shifts between synchronous and traveling-wave dynamics, primate-like circuits may preserve stable population synchrony while flexibly adjusting functional connectivity. However, this interpretation should be treated cautiously because the main species-level comparisons were based on the 7-s simulation window analyzed in our model. To further address whether late-stage activity could shift toward different propagation regimes, we extended the IN–TC inhibitory-connectivity sweep to 10-s trials. These longer simulations showed that late activity depended on the inhibitory parameter regime rather than following a single trajectory toward stronger synchrony or stronger propagation. Intermediate IN–TC scaling values supported broader recruitment and more synchronous activity, whereas excessive inhibitory scaling restricted recruitment and produced sparse activity. Thus, the extended trials do not support a simple interpretation in which primate-like closed-loop activity necessarily transitions toward traveling-wave-like propagation over time. Instead, they suggest that late-stage synchrony and propagation depend on the broader inhibitory state of the circuit. Future work should use explicit long duration simulations across additional loop configurations and complement PCA/MPT-based descriptors with correlation-based or phase-based synchrony measures to further test the robustness of this interpretation.

This divergence has important implications for interpreting species differences in spindle generation, sensory gating, and corticothalamic timing. Primate-like circuits may be tuned for more precise temporal coordination, while rodent-like circuits may be more prone to sequential or wave-like patterns that could be suited for different information processing.

### Common and divergent sleep spindle metrics in rodent-like and primate-like models

Sleep spindles arise from dynamic TC interactions, with spindle variability reflecting distinct network states [81]. In our simulations, the raster of spindles and power spectra revealed clear species differences in how these network dynamics manifested (Figures 6 and 7). Across all loop configurations, the rodent-like spindle patterns appeared more irregular and heterogeneous across core and matrix than those in the primate-like model. In the raster plots, rodent-like spindles showed more variation in the shape and width, as well as greater inconsistency in how many neurons participated from spindle to spindle. In contrast, the primate-like simulations displayed spindles that were more uniform, with more consistent spindle shapes and clearer boundaries across core and matrix.

In terms of frequency, PSD analysis showed that both rodent-like and primate-like models exhibited measurable spindle-band power, but that this power was organized differently across species and loop architectures (Figure 7; Table S8). Rather than showing a simple species-wide increase or decrease in power, the spectra revealed species- and architecture-dependent differences in peak frequency, 13–15 Hz band power, and normalized spectral contrast.

Taken together, observing both the raster plots and the power spectra indicate that rodent-like spindle activity is more variable and less consistent, whereas primate-like spindles are more coherent and rhythmically uniform. However, the PSD results suggest that this difference is best interpreted as a shift in spindle-band organization rather than a uniform difference in overall spectral power.

### Species- and loop-dependent differences in spindle-band signatures

The power spectrum of TC activity provided another source of insights regarding species-and loop-dependent specific dynamics [44]. Through the spectral analysis of TC activity, prior work has identified EEG “fingerprint” regions in humans/primates, which are specific frequency bands associated with functional Ctx specialization and statistically nonsignificant local field potential (LFP) “frequency bumps” in rodents [62]. Further research utilizing EEG/MEG metrics could help determine how these spectral differences relate to cognitive processing and disease states.

Motivated by this framework, we examined whether rodent-like and primate-like TC models showed comparable differences in the distribution of spindle-band power. In our analysis, we used PSD analysis to characterize continuous spectral structure across the 8–20 Hz range (Figure 7) and quantified power in lower and higher spindle-band ranges (Table S8).

Our results also revealed species- and loop-specific differences in TC spindle-band dynamics shaped by loop architecture. In open loop conditions, rodent-like and primate-like networks displayed similar general spectral power patterns. However, the clearest open loop difference was pathway-specific: primate-like matrix loops showed higher power in both the 10–12 and 13–15 Hz ranges, whereas core differences were less pronounced (Figure 7A–D). Thus, open-loop differences were quantitative and pathway-dependent rather than reflecting a frequency component present in one species but absent in the other.

In closed loop conditions, rodent-like circuits showed the strongest low-sigma power. The effect was most pronounced in the matrix configuration, where the rodent closed matrix configuration produced the largest 10–12 Hz power across conditions and a dominant peak near 11 Hz (Figure 7F). Rodent closed-loop core activity showed a similar but weaker pattern, with elevated 10–12 Hz power relative to 13–15 Hz power (Figure 7E). Primate-like circuits showed low-sigma activity, but they were less dominated by the 10–12 Hz band and a relatively greater contribution of 13–15 Hz power (Figure 7G and H). Overall, closed loop configurations showed stronger lower spindle range dominance in the rodent-like configuration and more balanced spindle-band profile in primate-like circuits.

In hybrid loop configurations, both rodent-like core and matrix pathways demonstrated higher absolute power in 10–12 and 13–15 ranges (Figure 7I and J), whereas primate-like hybrid pathways revealed lower absolute power but relatively higher fast/slow ratios (Figure 7K and L). This indicates that primate hybrid spectra were weaker overall but less dominated by the lower spindle power. Overall, hybrid loops therefore suggest differences in the relative balance of lower and higher spindle bands.

Together, these findings suggest that species-informed TC architecture shapes spindle-band dynamics by altering the balance of power across lower and higher spindle-band ranges. The strongest pattern was the concentration of power in the 10–12 Hz range in the rodent-like closed loop circuits, especially the matrix. Primate-like circuits did not show uniformly dominant fast-spindle peaks, but instead generally showed a more balanced distribution of power in the 10–12 and 13–15 Hz ranges. Thus, the model supports the idea that distinct species-specific TC architectures affect oscillatory dynamics, as reflected in species-specific differences in spindle-band power balance.

Prior empirical work shows that rodent sigma-band activity can be difficult to summarize as a single distinct peak: rodent EEG spectra may show a broad “shoulder” rather than a clear sigma peak, while local LFP recordings can reveal stronger area-specific sigma power and discrete spindle events [62]. In our model, however, rodent-like closed loop configurations, especially the matrix pathway, produced the strongest 10–12 Hz power and dominant low-sigma peak near 11 Hz. This suggests that the rodent-like TC loop architecture may strongly amplify lower spindle-band activity. Because our model included core–matrix mixing, this effect may also reflect interactions between core and matrix influences. Rodent-like TC loops may operate through a more integrated architecture, where core and matrix circuits blend and are not as segregated as they are in primates. Anatomical findings showing more integrated architecture or mixed core and matrix TC connectivity in rodents provide a plausible framework for this interpretation [82–84]. However, the current analysis does not directly isolate the contribution of core–matrix mixing. Future simulations that systematically vary the degree of core–matrix interactions would be needed to determine whether such mixing is necessary for the observed enhanced lower sigma bands. This supports the need to incorporate matrix influence when modeling rodent-like TC dynamics.

In primates, prior anatomical work supports clearer structural and functional specialization between core and matrix TC pathways [25]. In our model, the primate-like circuits showed a different spindle band signature from the rodent-like loops: primate-like circuits generally showed a more balanced distribution of power across the 10–12 Hz and 13–15 Hz ranges, especially when compared to fast/slow power ratios across matched rodent-like and primate-like conditions. Thus, rodent-like circuits showed stronger low-sigma dominance, while the primate-like loops showed a relatively greater contribution of higher spindle-band frequencies.

Hybrid loop configurations further supported this interpretation. Rodent-like hybrid core and matrix pathways showed higher absolute 10–12 and 13–15 power than in primate-like circuits. Primate-like hybrid spectra were weaker overall but showed relatively higher fast/slow ratios, indicating a more balanced distribution across lower and higher spindle ranges. Therefore, the hybrid loop results point to differences in spindle-band power balance across species, supporting the possibility that structural distinctions between species shape spindle expression across loop configurations [58–60].

Although we compare our in-silico findings to previous empirical studies, it is important to note that the data sources differ in recording techniques. Prior work has primarily relied on EEG and LFP recordings, which capture activity at different spatial and temporal scales. On the other hand, our model uses averaged neuronal activity as a simplified proxy to approximate electrophysiological signals. This presents a limitation of the present model interpretation: our simulated signals are not direct analogs of biological EEG or LFP recordings. Consequently, comparisons between our simulated power spectra and empirical EEG or LFP-based analysis [62] should be interpreted with caution.

### Expanding the role of the cortex and Ctx lamination in TC models

Beyond the IN and core–matrix dynamics, species differences in Ctx expansion and laminar organization play a crucial role in shaping TC interactions. Primate cortices exhibit greater differentiation in cytoarchitecture which facilitates more complex cortico–Ctx and cortico–thalamic feedback loops [11, 24, 25, 85]. This expanded Ctx hierarchy allows for more refined top-down regulation of thalamic activity, influencing synchrony and oscillation dynamics. Rodent cortices, on the other hand, have more compact and less stratified laminar structure, leading to differences in TC connectivity patterns. The relatively simpler Ctx organization in rodents likely results in more direct thalamic projections with less Ctx feedback [86, 87], making these circuits more sensitive to parameter-driven fluctuations in spindles and synchrony. Additionally, the limited presence of a well-developed granular level (L4) in certain Ctx levels in rodents may affect thalamic transmission, especially in sensory processing [88].

### Implications for modeling of neurodevelopmental disorders

Based on our findings, *in silico* models of the TC loop should incorporate IN and gradient-based associative structures to more accurately reflect species-specific dynamics. Interneuronal activity, which is prominent in primates but significantly lower in rodent thalamus, plays a crucial role in shaping spiking activity and network dynamics. These distinctions must be integrated into computational models to ensure realistic simulations of TC function. Furthermore, rodent models may exhibit a stronger influence of matrix-dominated pathways, potentially impacting TC circuit stability and power-frequency relationships.

Additionally, observed differences in TC synchrony and spindle dynamics between rodent-like and primate-like model configurations provide a valuable framework for understanding the role of TC dysfunction in neurodevelopmental and neuropsychiatric disorders, such as autism spectrum disorder (ASD) and SCZ [62, 89]. Differences in core–matrix dynamics and synchrony suggest that computational models must integrate mechanisms that account for gradual, spatially structured changes in connectivity and synaptic strength to capture these pathophysiological features. Future studies could utilize EEG or MEG metrics to validate these findings and refine disorder-specific models. Furthermore, although altered spindle activity has been reported in disorders such as ASD and SCZ, the present study does not directly model disease mechanisms. We therefore interpret the present framework as a platform for future mechanistic studies rather than as evidence for specific disorder pathophysiology. In future work, disorder-relevant perturbations could be introduced, for example by altering inhibitory conductances or spatial connectivity weights, and then testing whether the produced activity patterns reproduce spindle abnormalities documented in disease states.

## Supplementary Material

MS_BZ-NM-IR-AR_final_submission_SLEEP_Advances_supplement_zpag100(1)

## Data Availability

Data supporting the findings of this study are included in the manuscript and the Supplementary Materials. The code used for the simulations and analyses is available on GitHub at https://github.com/i-romanov/Biophysical-Modeling-of-Thalamocortical-Circuit-Dynamics.
