# Supplementary material for: Biophysical modeling of thalamocortical circuit dynamics: species-specific insights into neural synchrony, sleep spindles, and circuit mechanisms
Source: Sleep Adv. 2026 Sep 4;7(3):zpag100. doi: 10.1093/sleepadvances/zpag100 (PMC13615542; doi:10.1093/sleepadvances/zpag100)
Supplement: Supplementary file 1 [file ms_bz-nm-ir-ar_final_submission_sleep_advances_supplement_zpag100(1).docx]

**Biophysical Modeling of Thalamocortical Circuit Dynamics: Species-Specific Insights into Neural Synchrony, Sleep Spindles, and Circuit Mechanisms**

Basilis Zikopoulos^1, 2, 3, 4*^, Natalia Matuk^3,5^, Irina Romanova^3,5^, Arash Yazdanbakhsh^1,2,5*^

^1^Graduate Program for Neuroscience, Boston University, Boston, MA, United States

^2^Center for Systems Neuroscience, Boston University, Boston, MA, United States

^3^Human Systems Neuroscience Laboratory, Department of Health Sciences, Program in

Human Physiology, Boston University, Boston, MA, 02215 United States

^4^Department of Anatomy & Neurobiology, Boston University School of Medicine, Boston,

MA, United States

^5^Computational Neuroscience and Vision Laboratory, Department of Psychological and

Brain Sciences, Boston University, Boston, MA, 02215 United States

*Corresponding authors

⁘ Corresponding authors email addresses

Arash Yazdanbakhsh, (64 Cummington Mall, Department of Psychological & Brain

Sciences, Boston University, MA, 02215), Email: yazdan@bu.edu

Basilis Zikopoulos, (635 Commonwealth Avenue, Department of Health Sciences

Boston University, Boston MA 02215), Email: zikopoul@bu.edu

**Supplementary Tables:**

| Current | Neuron Types | Variable(s) | Function |
| --- | --- | --- | --- |
| I_Na+_ | TRN, TC, Ctx, IN | m, h | $\alpha_{m}=\frac{-0.32\left( V-V_{T}-13 \right)}{e^{-\frac{V-V_{T}-13}{4}}-1}$  $\beta_{m}=\frac{0.28\left( V-V_{T}-40 \right)}{e^{\frac{V-V_{T}-40}{5}}-1}$  $\alpha_{h}=0.128 e^{-\frac{V-V_{T}-17}{18}}$  $\beta_{h}=\frac{4}{1+e^{\frac{-V-V_{T}-40}{5}}}$ |
| I_K+_ | TRN, TC, Ctx, IN | n | $\alpha_{n}=\frac{-0.032\left( V-V_{T}-15 \right)}{e^{-\frac{V-V_{T}-15}{5}}-1}$  $\beta_{n}=0.5 e^{-\frac{V-V_{T}-10}{40}}$ |
| I_T_ | TRN | m, h | $m_{\infty}\left( V \right)=\frac{1}{1+e^{-\frac{V+52}{7.4}}}$  $\tau_{m}\left( V \right)=\frac{0.44+0.15}{e^{\frac{V+27}{10}}+e^{-\frac{V+102}{15}}}$  $h_{\infty}\left( V \right)=\frac{1}{1+e^{\frac{V+80}{5}}}$  $\tau_{h}\left( V \right)=\frac{22.7+0.27}{e^{\frac{V+48}{4}}+e^{-\frac{V+407}{50}}}$ |
| I_T_ | TC | m, h | $m_{\infty}\left( V \right)=\frac{1}{1+e^{-\frac{V+65}{7.8}}}$  $\tau_{m}\left( V \right)=0.15 m_{\infty}\left( V \right) \left( 1.7+e^{-\frac{V+30.8}{13.5}} \right)$  $\alpha_{1}\left( V \right)=\frac{e^{-\frac{V+162.3}{17.8}}}{0.26}$  $K\left( V \right)=\sqrt{0.25+e^{\frac{V+85.5}{6.3}}}-0.5$  $\tau_{2}\left( V \right)=\frac{62.4}{1+e^{\frac{V+39.4}{30}}}$  $\alpha_{2}\left( V \right)=\frac{1}{\tau_{2}\left( V \right) \backslash big\left( K\left( V \right)+1\backslash big \right)}$  $\alpha_{2}\left( V \right)=\frac{1}{\tau_{2}\left( V \right) \left( K\left( V \right)+1 \right)}$  $\frac{dd}{dt}=\alpha_{2}\left( V \right)\left( K\left( V \right) \left( 1-h-d \right)-d \right)$ |
| I_H_ | TC | S_1_, F_1_  S_2_, F_2_ | $H_{\infty}\left( V \right)=\frac{1}{1+e^{\frac{V+68.9}{6.5}}}$(S_1_ & F_1_)  $\tau_{S}\left( V \right)=e^{\frac{V+183.6}{15.24}}$  $\tau_{F}\left( V \right)=\frac{e^{\frac{V+158.6}{11.2}}}{1+e^{\frac{V+75}{5.5}}}$  $\alpha_{S}=\frac{H_{\infty}\left( V \right)}{\tau_{S}\left( V \right)}$  $\beta_{S}=\frac{1-H_{\infty}\left( V \right)}{\tau_{S}\left( V \right)}$  $\alpha_{F}=\frac{H_{\infty}\left( V \right)}{\tau_{F}\left( V \right)}$  $\beta_{F}=\frac{1-H_{\infty}\left( V \right)}{\tau_{F}\left( V \right)}$  $\frac{dS_{2}}{dt}=k_{2} \left( S_{2}-C S_{1} \right)$ |

**Supplementary Table S1**. Voltage-gated and Intrinsic Currents Functions. The gating and time-constant functions are listed for each current, with the corresponding neuron types in which they are expressed: I_Na+_ (fast sodium), I_K+_ (delayed rectifier potassium), I_T_ (T-type calcium), I_H_ (hyperpolarization-activated current).

| Neuron Type | Currents | $\vec{g}$  mS/cm^2^ | E_i_  (mV) | V_initial_ (mV) |
| --- | --- | --- | --- | --- |
| Cortex | I_Na_ | 50 | 50 | -65 |
|  | I_K_ | 5 | -90 |  |
|  | I_L_ | 0.1 | -70 |  |
| TRN | I_Na_ | 100 | 50 | -84 |
|  | I_K_ | 10 | -90 |  |
|  | I_L_ | 0.05 | -75 |  |
|  | I_T_ | 1.75 | 120 |  |
| TC | I_Na_ | 100 | 55 | -80 |
|  | I_K_ | 10 | -100 |  |
|  | I_L_ | 50 | -86 |  |
|  | I_H_ | 0.02 | -43 |  |
| IN | I_Na_ | 100 | 55 | -65 |
|  | I_K_ | 10 | -100 |  |
|  | I_L_ | 50 | -86 |  |

**Supplementary Table S2.** Neuron-Type Specific Constants. The maximal conductances ($\bar{g}$), reversal potentials (E_i_), and initial voltages (V_initial_) are listed for Cortex (Ctx), thalamic reticular nucleus (TRN), thalamocortical relay nurons (TC), and interneurons (IN).

| Current | **α** M^-1^S^-1^ | **β** S^-1^ | **Ei** (mV) |
| --- | --- | --- | --- |
| AMPA | 3.1842 | 0.1429 | 0 |
| GABA_A_ | 0.53 | 0.184 | -100 |

**Supplementary Table S3.** Ionotropic Synaptic Current Parameter Values. The forward (α) and backward (β) binding rates, and reversal potentials (E_j_), are listed for AMPA and GABA_A_ receptors.

| Metabotropic Currents | **K_1_**  M^-1^S^-1^ | **K_2_**  S^-1^ | **K_3_**  S^-1^ | **K_4_**  S^-1^ | **K_d_**  μM^2^ | **E_i_**  (mV) |
| --- | --- | --- | --- | --- | --- | --- |
| MGluR | 1.3 | 6.6 | 1.9 | 0.0064 | 100 | 0 |
| GABA_B_ | 1.3 | 6.6 | 0.9 | 0.0064 | 100 | -100 |

**Supplementary Table S4.** Metabotropic Synaptic Current Parameter Values. The rate constants for neurotransmitter binding (K_1_) and unbinding (K_2_), second-messenger sensitization (K_3_) and desensitization (K_4_), the dissociation constant (K_d_), and the reversal potentials (E_j_) are listed for MGluR and GABA_B_ are provided.

| **Source** | **Target** | **Pathway type** | **Receptor model** | **Spatial kernel** |
| --- | --- | --- | --- | --- |
| Core TC | Core TRN | within-loop | ionotropic AMPA | Core_DoG |
| Core TRN | Core TC | within-loop | GABA_A + GABA_B | Core_DoG |
| Core TC | Core Ctx | within-loop | ionotropic AMPA | Core_DoG |
| Core Ctx | Core TC | within-loop | metabotropic AMPA/mGluR-like | Core_DoG |
| Core Ctx | Core TRN | within-loop | metabotropic AMPA/mGluR-like | Core_DoG |
| Matrix TC | Matrix TRN | within-loop | ionotropic AMPA | Matrix_DoG |
| Matrix TRN | Matrix TC | within-loop | GABA_A + GABA_B | Matrix_DoG |
| Matrix TC | Matrix Ctx | within-loop | metabotropic AMPA/mGluR-like | Matrix_DoG |
| Matrix Ctx | Matrix TC | within-loop | ionotropic AMPA | Matrix_DoG |
| Matrix Ctx | Matrix TRN | within-loop | ionotropic AMPA | Matrix_DoG |
| Matrix Ctx | Core Ctx | cortico-cortical mixing | ionotropic AMPA | Core_DoG |
| Core Ctx | Matrix Ctx | cortico-cortical mixing | ionotropic AMPA | Matrix_DoG |

**Supplementary Table S5. Projection mapping in the thalamocortical model.** Projection-by-projection summary of source and target populations, pathway class, receptor model, and the spatial kernel applied to each connection

| **Pathway type** | **Kernel support** | **Excitatory Gaussian** | **Inhibitory Gaussian** | **Kernel definition** |
| --- | --- | --- | --- | --- |
| Core | 1 × 7 | (σ = 0.875) | (σ = 1.75) | $DoG =G_{ex} - 0.5 G_{inh}$ |
| Matrix | 1 × 29 | (σ = 3.625) | (σ = 7.25) | $DoG =G_{ex} - 0.5 G_{inh}$ |

**Supplementary Table S6. Spatial kernel definitions for core and matrix projections.**
Parameters of the one-dimensional difference-of-Gaussians (DoG) kernels used to model spatial spread across the neuron axis. Kernel support refers to the size of the one-dimensional spatial filter applied across the neuron axis. Accordingly, core pathways used a narrower local kernel (1 × 7), whereas matrix pathways used a broader kernel (1 × 29).

| **Model** | **Pathway** | **Population** | **Mean participating neurons** | **Mean participation fraction** | **Mean event rate in burst window (Hz)** | **Mean peak active fraction** |
| --- | --- | --- | --- | --- | --- | --- |
| Primate closed | Core | Cortex | 77.50 | 0.7750 | 22.90 | 0.0975 |
| Primate closed | Core | IN | 74.50 | 0.7450 | 7.73 | 0.0600 |
| Primate closed | Core | TRN | 72.75 | 0.7275 | 15.16 | 0.0975 |
| Primate closed | Core | TC | 25.50 | 0.2550 | 0.46 | 0.0200 |
| Primate closed | Matrix | Cortex | 85.00 | 0.8500 | 86.56 | 0.1650 |
| Primate closed | Matrix | IN | 76.50 | 0.7650 | 14.57 | 0.0875 |
| Primate closed | Matrix | TRN | 80.75 | 0.8075 | 16.88 | 0.0975 |
| Primate closed | Matrix | TC | 32.25 | 0.3225 | 0.57 | 0.0350 |
| Rodent hybrid | Core | Cortex | 51.50 | 0.3433 | 4.40 | 0.0350 |
| Rodent hybrid | Core | TRN | 43.75 | 0.2917 | 5.48 | 0.0400 |
| Rodent hybrid | Core | TC | 23.25 | 0.1550 | 0.32 | 0.0133 |
| Rodent hybrid | Matrix | Cortex | 80.50 | 0.5367 | 64.47 | 0.0850 |
| Rodent hybrid | Matrix | TRN | 78.25 | 0.5217 | 10.37 | 0.0633 |
| Rodent hybrid | Matrix | TC | 41.25 | 0.2750 | 0.45 | 0.0283 |

**Supplementary Table S7. Burst-window activation summaries for representative spatiotemporal rasters.** For the representative primate closed-loop and rodent hybrid-loop examples shown in Supplementary Figures S6 and S5, respectively, we quantified burst-window participation using the same threshold criterion used to generate the raster plots. Participating neurons were defined as neurons exceeding threshold at least once within a given burst window. Reported values are averaged across the identified burst windows for each population and include the mean number of participating neurons, mean participation fraction, mean threshold-crossing event rate within burst windows, and mean peak active fraction.

| **Loop** | **Species** | **Pathway** | **10–12 Hz power (μV²)** | **13–15 Hz power (μV²)** | **Fast/slow ratio** | **Peak frequency (Hz)** |
| --- | --- | --- | --- | --- | --- | --- |
| Open | Rodent | Core | 143.46 ± 39.12 | 105.77 ± 38.74 | 0.71 ± 0.09 | 13.3 ± 1.2 |
|  |  | Matrix | 185.29 ± 55.90 | 101.76 ± 32.96 | 0.55 ± 0.03 | 12.1 ± 0.9 |
|  | Primate | Core | 189.18 ± 77.24 | 131.35 ± 48.93 | 0.79 ± 0.29 | 11.3 ± 0.6 |
|  |  | Matrix | 366.31 ± 136.08 | 286.62 ± 134.16 | 0.65 ± 0.12 | 12.5 ± 1.0 |
| Closed | Rodent | Core | 785.93 ± 273.06 | 101.21 ± 28.02 | 0.23 ± 0.08 | 11.3 ± 0.2 |
|  |  | Matrix | 2379.66 ± 1038.37 | 147.33 ± 30.31 | 0.15 ± 0.07 | 10.9 ± 0.2 |
|  | Primate | Core | 307.26 ± 128.63 | 154.21 ± 72.92 | 0.48 ± 0.12 | 10.5 ± 0.1 |
|  |  | Matrix | 592.81 ± 268.31 | 294.45 ± 121.25 | 0.49 ± 0.10 | 10.6 ± 0.1 |
| Hybrid | Rodent | Core | 309.84 ± 108.34 | 104.63 ± 37.91 | 0.35 ± 0.07 | 11.5 ± 1.0 |
|  |  | Matrix | 489.21 ± 195.06 | 166.12 ± 65.19 | 0.34 ± 0.05 | 10.8 ± 0.4 |
|  | Primate | Core | 135.10 ± 62.03 | 63.99 ± 20.88 | 0.56 ± 0.05 | 10.2 ± 0.1 |
|  |  | Matrix | 249.23 ± 107.62 | 128.25 ± 51.25 | 0.55 ± 0.03 | 11.2 ± 0.8 |

**Supplementary Table S8. Quantitative PSD summaries across thalamocortical loop configurations. Power was quantified in the slow spindle-band range (10–12 Hz) and fast spindle-band range (13–15 Hz). Fast/slow ratio was defined as 13–15 Hz power divided by 10–12 Hz power. Peak frequency indicates the most prominent PSD peak within the 8–20 Hz analyzed range. Values are reported as mean ± SEM across five cortical subpopulation blocks per condition.**

| Model | Axis | Connection | Scaling factor (sf) | Initial value | Control value |
| --- | --- | --- | --- | --- | --- |
| Rodent | x-axis | Core TC→Ctx | Core_sfAMPA_TC_Ctx | 0.00001 | 0.5 |
| Rodent | y-axis | Matrix TC→Ctx | Matrix_sfAMPAm_TC_Ctx | 1 | 45 |
| Rodent | z-axis | Core→Matrix cortical mixing | Core_sfAMPA_MatrixCtx_CoreCtx | 0.01 | 0.12 |
| Rodent | z-axis | Matrix→Core cortical mixing | Matrix_sfAMPA_CoreCtx_MatrixCtx | 0.00001 | 0.18 |
| Primate | x-axis | Core TC→Ctx | Core_sfAMPA_TC_Ctx | 0.5 | 0.5 |
| Primate | y-axis | Matrix TC→Ctx | Matrix_sfAMPAm_TC_Ctx | 1 | 45 |
| Primate | z-axis | Core→Matrix cortical mixing | Core_sfAMPA_MatrixCtx_CoreCtx | 0.01 | 0.12 |
| Primate | z-axis | Matrix→Core cortical mixing | Matrix_sfAMPA_CoreCtx_MatrixCtx | 0.00001 | 0.18 |

**Supplementary Table S9. Initial and control values for parameter-cube axes.** The x-, y-, and z-axes correspond to core thalamocortical drive, matrix thalamocortical drive, and cortico-cortical core–matrix mixing, respectively. Values indicate the initial and control conductance-scaling factors used for the rodent-like and primate-like model parameter scans.

S**upplementary Figures:**

**Supplementary Figure S1. Single-cell validation traces under current clamp**. Representative membrane-potential responses of isolated model neurons to hyperpolarizing and depolarizing current steps are shown for cortical (A,B), TRN (C,D), TC (E,F), and IN (G,H) cell models. These simulations use the same intrinsic membrane and gating equations as the network model, with synaptic coupling removed to isolate cell-intrinsic behavior. TRN and TC traces confirm expected low-threshold dynamics and rebound behavior, while cortical and IN traces confirm spiking responses under depolarizing drive.

**Supplementary Figure S2. Rodent-like and Primate-like Closed Thalamocortical Loop Sleep Spindles**. A. Representative examples of core, matrix and mixed sleep spindles in the rodent-like and primate-like closed loop model, with detected spindle events marked in red. B. Boxplots of spindle amplitude, duration, and density for core, matrix, and mixed signals across species. C. Histograms of rodent-like spindle amplitude, duration, and density for core, matrix, and mixed signals. D. Histograms of primate-like spindle amplitude, duration, and density for core, matrix, and mixed signals.

**Supplementary Figure S3. Rodent-like and Primate-like Open Thalamocortical Loop Sleep Spindles**. A. Representative examples of core, matrix and mixed sleep spindles in the rodent-like and primate-like open loop model, with detected spindle events marked in red. B. Boxplots of spindle amplitude, duration, and density for core, matrix, and mixed signals across species. C. Histograms of rodent-like spindle amplitude, duration, and density for core, matrix, and mixed signals. D. Histograms of primate-like spindle amplitude, duration, and density for core, matrix, and mixed signals.

**Supplementary Figure S4. Rodent-like and Primate-like Hybrid Thalamocortical Loop Sleep Spindles**. A. Representative examples of core, matrix and mixed sleep spindles in the rodent-like and primate-like hybrid loop model, with detected spindle events marked in red. B. Boxplots of spindle amplitude, duration, and density for core, matrix, and mixed signals across species. C. Histograms of rodent-like spindle amplitude, duration, and density for core, matrix, and mixed signals. D. Histograms of primate-like spindle amplitude, duration, and density for core, matrix, and mixed signals.

**Supplementary Figure S5. Representative spatiotemporal rasters across cortical and thalamic populations in the rodent-like hybrid-loop model.** Representative spatiotemporal rasters are shown for cortex (A,D), TRN (B,E), and TC (C,F) populations in the rodent-like hybrid-loop model. Core pathway activity is shown in the top row (A–C) and matrix pathway activity in the bottom row (D–F). These panels illustrate population-specific differences in synchrony and propagation across circuit nodes under the hybrid-loop configuration.

**Supplementary Figure S6. Representative spatiotemporal rasters across cortical and thalamic populations in the primate-like closed-loop model.** Representative spatiotemporal rasters are shown for cortex (A,E), IN (B,F), TRN (C,G), and TC (D,H) populations in the primate-like closed-loop model. Core pathway activity is shown in the top row (A–D) and matrix pathway activity in the bottom row (E–H). These panels illustrate population-specific differences in synchrony and propagation across cortical and thalamic nodes under the closed-loop configuration.

**Supplementary Figure S7.** **PCA-based quantification of representative non-control primate-like cortical activity bands.** Representative raster plots are shown for core (A,C) and matrix (B,D) cortical activity in the primate-like model at a non-control parameterization. Closed-loop activity is shown in the top row (A,B) and open-loop activity in the bottom row (C,D). For each analyzed activity band, burst centers of mass are marked with dots and the PCA-derived eigenvector is overlaid. Deviation-from-vertical angle θ and mean propagation time, MPT (ms/neuron), are reported for each activity band.

**Supplementary Figure S8. Representative spindle-like event detection within a zoomed cortical trace.** A zoomed-in segment from the rodent-like closed-loop matrix condition is shown. A. Unfiltered averaged cortical signal. B. Corresponding 9–13 Hz bandpass-filtered signal. C. Z-scored amplitude envelope used for event detection. Red dashed vertical lines mark event onset and offset, and the horizontal dashed line marks the detection threshold. The raw and filtered traces are shown on different y-axis scales to make the smaller, filtered spindle-band component visible relative to the larger unfiltered voltage signal.
